# Investigating intraspecific variability in the biological responses of sea urchins *(Paracentrotus lividus)* to seawater acidification

**DOI:** 10.1007/s11356-024-34618-7

**Published:** 2024-08-09

**Authors:** Davide Asnicar, Federica Stranci, Silvia Monti, Denis Badocco, Tihana Marčeta, Marco Munari, Maria Gabriella Marin

**Affiliations:** 1 Department of Biology, University of Padova, Via U. Bassi 58/B, 35131 Padova, Italy; 2 Aquatic Biosciences, Huntsman Marine Science Centre, 1 Lower Campus Road, E5B 2L7, St. Andrews, New Brunswick Canada; 3 Department of Chemical Sciences, University of Padova, Via Marzolo 1, 35131 Padova, Italy; 4 Institute of Marine Sciences (ISMAR), CNR, Castello 2737/F, 30122 Venezia, Italy; 5 Department of Integrative Marine Ecology, Fano Marine Centre, Stazione Zoologica Anton Dohrn, Fano, Italy

**Keywords:** Sea urchin, Seawater acidification, Biomarkers, Oxidative stress, Immune parameters, Gonadosomatic index, Intraspecific variability

## Abstract

Alterations in seawater chemistry posed by acidification may lead to immunological and antioxidant defence impairment in sea urchins, with differences among local populations. Here, we analyzed the effects of reduced pH on *Paracentrotus lividus*, with a multibiomarker approach, and the possible intraspecific variations in sea urchin responses. Two groups of animals with different ecological histories (i.e., the pattern of environmental characteristics and pressures experienced throughout the organism’s lifetime) were maintained at ambient pH and pH reduced of 0.4 units for 8 months. Changes in gonadosomatic index (GSI), immunological, and oxidative stress biomarkers were assessed in coelomic fluid, gonads, and digestive tract. Animals maintained at reduced pH showed limited impact of seawater acidification compared to the ambient pH condition. However, sea urchins from the two sites were differently influenced by the seawater pH (as shown by multivariate analyses). GSI and immunological and antioxidant status were differentially modulated between the two sexes, with generally higher values in females, but differences between sexes in relation to the pH of exposure were limited. Overall, our findings highlight that the impact of environmental stressors may differ in sea urchins from different locations. This has implications for the maintenance of *P. lividus* wild populations under future global change scenarios.

**Supplementary Information:**

The online version contains supplementary material available at 10.1007/s11356-024-34618-7.

## Introduction

The increased CO_2_ concentration in the surface seawater is causing the lowering of pH, a phenomenon called ocean acidification (OA) (Doney et al. 2009; Raven et al. 2005; Sabine et al. 2004). If compared with pH levels at the beginning of the twentieth century (pH ≈8.2), the seawater has already acidified by about 0.02 units per decade and a further reduction of 0.3-0.4 units is expected by the end of the century in a business-as-usual scenario (pH 7.8–7.7; Representative Concentration Pathway 8.5) (Fransner et al. 2022; IPCC 2019; Orr et al. 2005).

It has been tested that, especially in marine calcifying invertebrates, seawater acidification due to increase of CO_2_ content can disturb various processes including acid–base regulation and calcification (Byrne et al. 2011; Holtmann et al. 2013; Migliaccio et al. 2019), and cause alterations in immune-related variables (Castillo et al. 2017; Munari et al. 2019, 2020a; Sun et al. 2017; Wang et al. 2016), antioxidant defence (Moreira et al. 2016; Munari et al. 2018; Sui et al. 2017), growth (Bressan et al. 2014; Kamya et al. 2016; Munari et al. 2016; Waldbusser et al. 2015), physiological performance (Asnicar et al. 2021; Munari et al. 2020b), and reproduction (Cao et al. 2015; Lucey et al. 2015; Munari et al. 2022).

Among calcifiers, sea urchins, recognized to be keystone species in many habitats worldwide, have been used as model species in several studies to assess the extent of seawater acidification effects (for a review on the topic see Asnicar and Marin 2022). Attention has been recently paid to the potential impairment of sea urchin immune surveillance under seawater acidification conditions, due to disruption in coelomocyte functions. Indeed, coelomocytes are responsible for the main defence against pathogens and foreign materials (Endean 1966), thus influencing the health status of animals with cascading effects at the population and ecosystem levels.

Reduced pH could not only affect the immune response capability (Dupont and Thorndyke 2012; Marčeta et al. 2020; Migliaccio et al. 2019) but also promote oxidative stress and damage in tissues, as a consequence of acid–base disruption (Anand et al. 2021; Marčeta et al. 2020). An increase in reactive oxygen species (ROS) not properly counteracted by antioxidant defences ultimately ends up harming metabolic enzyme activities, ATP production, protein structure, and cellular membrane integrity, promoting apoptotic events (Lesser 2006). On the other hand, several studies showed that prolonged exposure to seawater acidification can lead to acclimation in sea urchins, with potential transgenerational benefits (Asnicar et al. 2022; Hoshijima and Hofmann 2019; Karelitz et al. 2019; Marčeta et al. 2022). However, knowledge of the effects of seawater acidification on sea urchin immunological and oxidative stress–related variables in long-term exposure is still scarce and need further investigation.

Therefore, this work aimed to evaluate potential differences in gonadal growth, immunological and biochemical endpoints of the sea urchin *Paracentrotus lividus* maintained at reduced pH conditions predicted for the end of this century (− 0.4 pH units; IPCC scenario RCP8.5; IPCC 2014) after a long-term exposure (8 months).

Since other studies highlighted that acidification can decrease the gonad index (Dworjanyn and Byrne 2018), the readiness to reproduce and gonad quality was evaluated through the gonadosomatic index in sea urchins reared at both pH levels.

The maintenance at lower pH conditions was demonstrated to impact sea urchins’ cell homeostasis and functionality, altering immunological and antioxidant status in short- to mid-term exposures and field studies (Anand et al. 2021; Dupont and Thorndyke 2012; Gattuso and Hansson, 2011; Marčeta et al. 2020; Migliaccio et al. 2019). A weakened defence system could result in higher susceptibility to other stressors (diseases, pollution, or toxins from harmful algal blooms) with further consequences for natural population maintenance (Bisanti et al. 2024; Lewis et al. 2016). Therefore, after long-term exposure, we used a multibiomarker approach to investigate immunological and antioxidant responses in coelomic fluid, gonad, and digestive tract of the sea urchins.

The battery of 12 biomarkers selected concerns immunological surveillance (coelomocyte characteristics, cytotoxicity, acid phosphatase, and lysozyme activity), antioxidant defences (the activity of superoxide-dismutase SOD, catalase CAT, and glutathione reductase GR enzymes), detoxification (the activity of glutathione-S-transferase GST enzyme), as well as oxidative damage (protein carbonyl content PCC and lipid peroxidation LPO) to check for alterations in proteins and lipids which can compromise cell membrane stability and tissue integrity.

Furthermore, the response to potential stressors, such as reduced pH, showed intraspecific differences due to the local biological adaptation of animals to the variability of seawater parameters (Asnicar et al. 2021; Thor et al. 2018; Vargas et al. 2017, 2022). Latitudinal variability in the susceptibility to pH reduction was highlighted in marine invertebrates, possibly related to differences in metabolic compensation mechanisms (Calosi et al. 2017; Thor and Oliva 2015; Asnicar and Marin 2022).

Responses to reduced pH can also vary between relatively close sites with different environmental characteristics (Conradi et al. 2019). For example, behavioral and metabolic responses to low pH were shown to vary in sea urchins collected from a lagoon and a coastal area and thus subjected to different variations in seawater parameters (Asnicar et al. 2021). Therefore, assessing biological responses to reduced seawater pH in sea urchins from different environments becomes crucial.

In this perspective, we performed parallel exposures with two groups of sea urchins with a different ecological history (i.e., the pattern of environmental characteristics and pressures experienced throughout their lifetime) to shed light on the extent, variability, and predictability of reduced pH impacts. One sampling site was selected in the Lagoon of Venice (lagoon site LS; 45° 13′ 41′′ N, 12° 16′ 13′′ E, sampled in July 2018), which is the largest lagoon of the Mediterranean Sea and shows wide fluctuations of seawater parameters due to its shallowness, the influence of freshwater inputs and exchange with the Adriatic Sea. The other sampling site was selected in a more stable environment in the Northern Adriatic Sea, far from remarkable anthropogenic activities and riverine influences (marine site MS; 45° 43′ 30′′ N, 13° 41′ 31′′ E, sampled in July 2019).

The expected result is that *P. lividus* from the highly variable environment (the lagoon) would be able to acclimate to the 0.4 pH units reduction, while *P. lividus* from the more stable environment (marine site) would present differences between pH conditions tested.

This study is part of a broader project aimed at identifying the potential effects of long-term exposure to seawater acidification on adult *P. lividus* by integrating multiple aspects at the biochemical, cellular, physiological, behavioral, and reproductive levels (Asnicar et al. 2021, 2022). Since this species is harvested and cultured for human consumption (Boudouresque and Verlaque 2020; Sartori et al. 2016), understanding the impact of future global changes on the physiological status of different populations of *P. lividus* could provide insights into the future feasibility of culture systems (FAO 2021).

## Materials and methods

### Sea urchin collection and exposure

In order to test the influence of the native environment on the response *of P. lividus* to a global change scenario, two experiments were carried out with animals collected in different locations. The two sites (LS and MS) were selected based on the different physicochemical variability of the areas (see details above). Details about the physicochemical parameters of the two sites have already been reported by Asnicar et al. (2021) and are additionally provided in Supplementary Material (Figs S1, S2, Tables S1, S2).

At each sampling site, sea urchins were collected by SCUBA divers at approximately 0.5–2 m depth. Animals were transferred in cooling boxes to the Hydrobiological Station (Department of Biology, University of Padova) in Chioggia. Upon arrival, the animals were acclimated to the laboratory conditions (temperature 26.26 ± 0.59 °C, salinity 34.14 ± 0.42, pH_T_ 8.18 ± 0.06; mean ± SD) for 1 week before the start of the experiment. The experiment consisted of a period of starvation followed by exposure to reduced pH. The starvation period (based on the method described by Grosjean et al. 1998 and Shpigel et al. 2004) was performed to ensure the atrophy of the gonads, and to start the exposure at the same gametogenic stage for all urchins from both sites (see Asnicar et al. 2021 for details).

Thereafter, the exposure to different pH conditions started: ambient pH (pH Amb) and pH reduced by 0.4 units (pH − 0.4; based on IPCC RCP8.5 scenario). pH levels were manipulated by bubbling CO_2_ and adjusted using a pH-controlling setup (ACQ110 Aquarium Controller Evolution, Aquatronica). Target pH was achieved by decreasing its value at a rate of 0.1 pH unit per day.

Sea urchins were maintained in 60 L aquaria with aerated seawater, with a photoperiod of 14:10 (light:dark), and fed ad libitum with spinach and corn (a low-cost, effective, and easy-to-standardize diet for urchins; see Sartori et al. 2016). For each experimental condition, three tanks per pH condition were prepared. Animals were distributed randomly and equally into the six experimental tanks (~ 28 specimens per tank for LS; ~ 31 for MS). Abiotic variables in the six tanks were checked daily using a multiparameter probe (Hi 9829, Hanna Instruments Italia Srl, Italy) and a benchtop pH meter (Basic 20, Crison, Spain) calibrated daily with Crison buffer solutions. The pH values throughout the experiments are reported on the total scale (pH_T_) (see Supplementary Material, Table S3). The pH obtained with the Crison NIST buffers was converted into the pH_T_ scale using the conversion equation described in the work of Badocco et al. (2021). The validity of the conversion equation was recently confirmed with independent measurements performed with a colorimetric pH sensor by Pastore et al. (2024).

Total alkalinity (TA) was determined via potentiometric titration using an automatic titrator (836 Titrando, Metrohm). Each seawater sample was thermostated at 25 °C (HAAKE C25P Phoenix II, ENCO) before titration. The titrations were performed in duplicate, and when the standard deviation of the duplicate test exceeded the threshold value of 3%, a third titration was performed.

The dissolved inorganic carbon (DIC) content, carbonate concentration values, and CO_2_ partial pressure (*p*CO_2_) in seawater were computed at the sampling temperature using the TA values. The application of the error propagation law allowed the computation of the standard deviations of the DIC, CO_3_ − , and *p*CO2 values. All thermodynamic equilibrium constants were computed according to Millero (1995 and 2006).

The maintenance of the animals in the laboratory setup has been previously described thoroughly in our companion paper (Asnicar et al. 2021).

### Tissue collection

After 8 months of exposure, coelomic fluid (CF) and tissues were sampled from 15 LS sea urchins and 25 MS sea urchins per experimental conditions. Differences in the number of animals sampled are due to differences in the starting number of animals. Mortality occurred but was not different between sites or pH levels (LS pH Amb 6.0%, pH − 0.4, 3.0%; MS pH Amb 3.2%, pH − 0.4, 7.5%).

Each specimen was measured (test diameter without spines) using a caliper (precision 0.01 cm) and weighted (Table 1). CF was collected from each animal with a sterile plastic syringe through the peristomal membrane, placed in 15-mL tubes, and stored on ice until analysis or subsequent treatment.

**Table 1:** Test diameter and weight of sampled animals expressed as mean ± standard deviation

Site	pH	Sex	N	Diameter (cm)	Wet weight (g)
LS	pH Amb	Female	7	4.03 ± 0.46	33.52 ± 11.38
		Male	8	4.47 ± 0.46	45.04 ± 12.77
	pH − 0.4	Female	9	4.10 ± 0.39	34.64 ± 10.07
		Male	6	4.12 ± 0.63	35.65 ± 17.23
MS	pH Amb	Female	12	4.66 ± 0.36	52.42 ± 11.56
		Male	13	4.70 ± 0.38	52.39 ± 11.62
	pH − 0.4	Female	13	4.55 ± 0.27	43.33 ± 6.90
		Male	12	4.57 ± 0.30	43.14 ± 8.81

Thereafter, the animal was opened with a scissor and the digestive tract and the gonads were excised. Tissues were maintained on ice, divided into aliquots, snap-frozen in liquid nitrogen, and stored at − 80 °C until processing.

One of the five gonads sampled was weighed and the gonadal-somatic index (GSI) was calculated as follows: [(gonad wet weight*5)/animal wet weight] *100 (Régis 1979). An aliquot of gonads was used to check the sex of the animals by a smear on a slide, checking for the presence of sperm or eggs under a microscope (Leica DM 750).

### Immunological biomarkers

An aliquot of CF was used immediately to measure total coelomocyte count (TCC), coelomocyte diameter and volume, and LDH activity. The remaining aliquots were centrifuged for 10 min at 3000 × *g* and 4 °C to separate coelomocytes and coelomic fluid without cells. Both coelomocyte and cell-free coelomic fluid (CFCF) aliquots were snap-frozen in liquid nitrogen and stored at − 80 °C until analyses.

TCC and coelomocyte diameter and volume were assessed with a Scepter™ 2.0 Automated Cell Counter (Millipore). A volume of 20 μL of coelomic fluid was diluted in 2 mL of Coulter Isoton II diluent. The TCC was expressed as the number of coelomocytes/mL of CF, while cell diameter and volume were expressed in μm and picolitres (pL), respectively.

For LDH assay, 700 µL of CF were centrifuged as reported above. A commercial kit (Cytotoxicity Detection Kit, Roche) was used to measure LDH (a proxy of cellular cytotoxicity quantification) in 500 µL of CFCF using a spectrophotometer (Beckman Coulter, DU 730). The results were expressed as optic density (OD) at 490 nm.

Lysozyme activity in coelomocytes and CFCF was measured spectrophotometrically as described in Matozzo et al. (2012a). The results were expressed as μg lysozyme/mg of protein. Protein concentration was quantified according to the method of Bradford (1976).

A commercial kit (Acid Phosphatase Assay Kit, Sigma-Aldrich) was also used to measure PHO activity spectrophotometrically in both coelomocytes and CFCF, following the manufacturer’s instructions. The results were expressed as U/mg protein. One unit of acid phosphatase was defined as the amount of enzyme that hydrolyses 1 μmol of 4-nitrophenyl phosphate/min at 4.8 pH and 37 °C.

### Oxidative stress biomarkers

Aliquots of digestive tracts and gonads were homogenized on ice using an Ultra-Turrax homogenizer (model T8 basic, IKA) with 1.5 mL of homogenization buffer (10 mM Tris–HCl, pH 7.5, with 0.15 M KCl, 0.5 M sucrose, 1 mM EDTA, and protease inhibitor cocktail; Sigma-Aldrich). Subsequently, samples were centrifuged at 12,000 g for 30 min at 4 °C (Eppendorf centrifuge model 5415R). Analyses were performed using the supernatants (SN). Protein concentrations in SN were quantified according to Bradford (1976). All biomarkers were measured spectrophotometrically and in triplicates.

SOD activity was measured using the xanthine oxidase/cytochrome c method proposed by Crapo and colleagues (Crapo et al. 1978). Enzyme activity was expressed as enzymatic units (U)/mg protein; one U SOD was defined as the sample’s amount causing 50% inhibition under the assay conditions.

CAT activity was measured according to the method of Aebi (1984) and expressed as U/mg protein. One unit of CAT was defined as the amount of enzyme that catalyzed the dismutation of 1 μmol of H_2_O_2_/min.

To measure GR activity, the method proposed by Smith and colleagues was used (Smith et al. 1988). The results were expressed as U/mg protein, with one U GR defined as the enzyme quantity causing the reduction of 1 µmol of DTNB to TNB at 25 °C, pH 7.5.

The activity of GST was determined following the method described by Habig and colleagues (Habig et al. 1974). The results were expressed in nmol/min/mg protein.

PCC was measured via the formation of labelled protein hydrazone derivatives, after 2,4-dinitrophenyl hydrazine (DNPH) reaction, and quantified at the spectrophotometer (Dalle-Donne et al. 2003, Mecocci et al. 1999). The carbonyl content was calculated from the SN absorbance via the molar absorption coefficient of 22,000 mol/cm and expressed as nmol/mg protein.

LPO was measured only in gonads due to a shortage of digestive tract material. Peroxidation of lipids was quantified using the malondialdehyde (MDA) assay described by Buege and Aust (1978) and results were expressed as nmoles of thiobarbituric reactive substances (TBARS)/mg protein (Csallany et al. 1984; Damiens et al. 2007).

All assays performed in this study have previously been validated (Marčeta et al. 2020; Marisa et al. 2016; Matozzo et al. 2012b).

### Statistical analysis

To test if differences in immunological and oxidative-stress variables analyzed were due to animal size, a non-parametric permutational multivariate analysis of variance (PERMANOVA, with 9999 permutations) was performed, applying the Euclidean distance matrix to the raw data, and using the urchins’ “diameter” as a factor. Results highlighted no statistical relationship with animal diameter (*R*^2^ = 0.897, F. model = 1.7192, p = 0.125); hence, the factor size was excluded from the following analysis.

To test the hypothesis that site (LS or MS), pH level (Amb or − 0.4), and sex had a significant influence on immunological and oxidative-stress variables, a three-factorial non-parametric permutational multivariate analysis of variance (PERMANOVA, with 9999 permutations) was performed, applying the Euclidean distance matrix to the raw data.

Additionally, a mixed linear model was used to highlight the effects of “site of origin,” “pH level,” and “sea urchin sex” and their interactions on each biomarker singularly, using “tank” as a random effect. Normality and homoscedasticity assumptions were checked on the data using Shapiro–Wilk’s and Bartlett’s tests, respectively. Since ANOVA assumptions were violated, a chi-squared (*Χ*^2^) distribution was applied to establish the statistical significance of the factors and their interaction. The mixed linear model was followed by a post hoc test (Tukey HSD test with Bonferroni correction) for pairwise comparisons between experimental conditions within each biomarker.

The differences between “sites,” “pH levels,” and “sexes” were visualized with a canonical correlation analysis (CCA) performed on the whole biomarker dataset.

Results are expressed as the mean ± standard error of the mean (SE). The software R and R Studio (R Core Team 2021) with the “r41sqrt10” package (Finos 2020) and the “vegan” package (Oksanen et al. 2019) were used.

## Results

### PERMANOVA

**Fig. 1:**
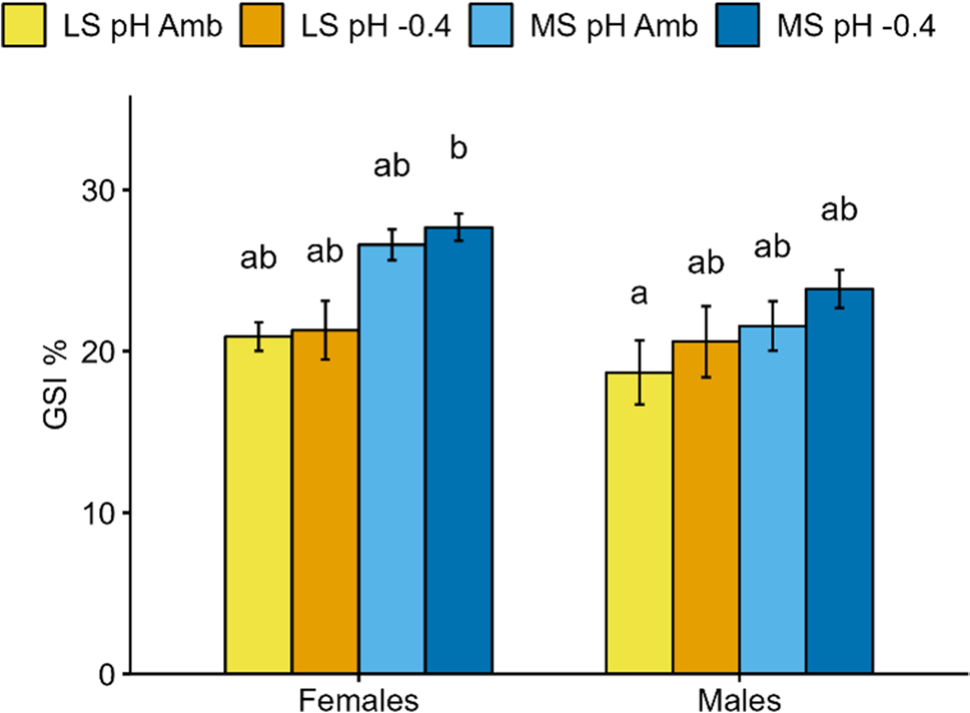
GSI results in *P. lividus* from different sites (LS, lagoon site; MS, marine site) maintained for 8 months at two pH levels (pH Amb and pH − 0.4). Different letters indicate significant differences among all conditions (*p* < 0.05)

**Fig. 2:**
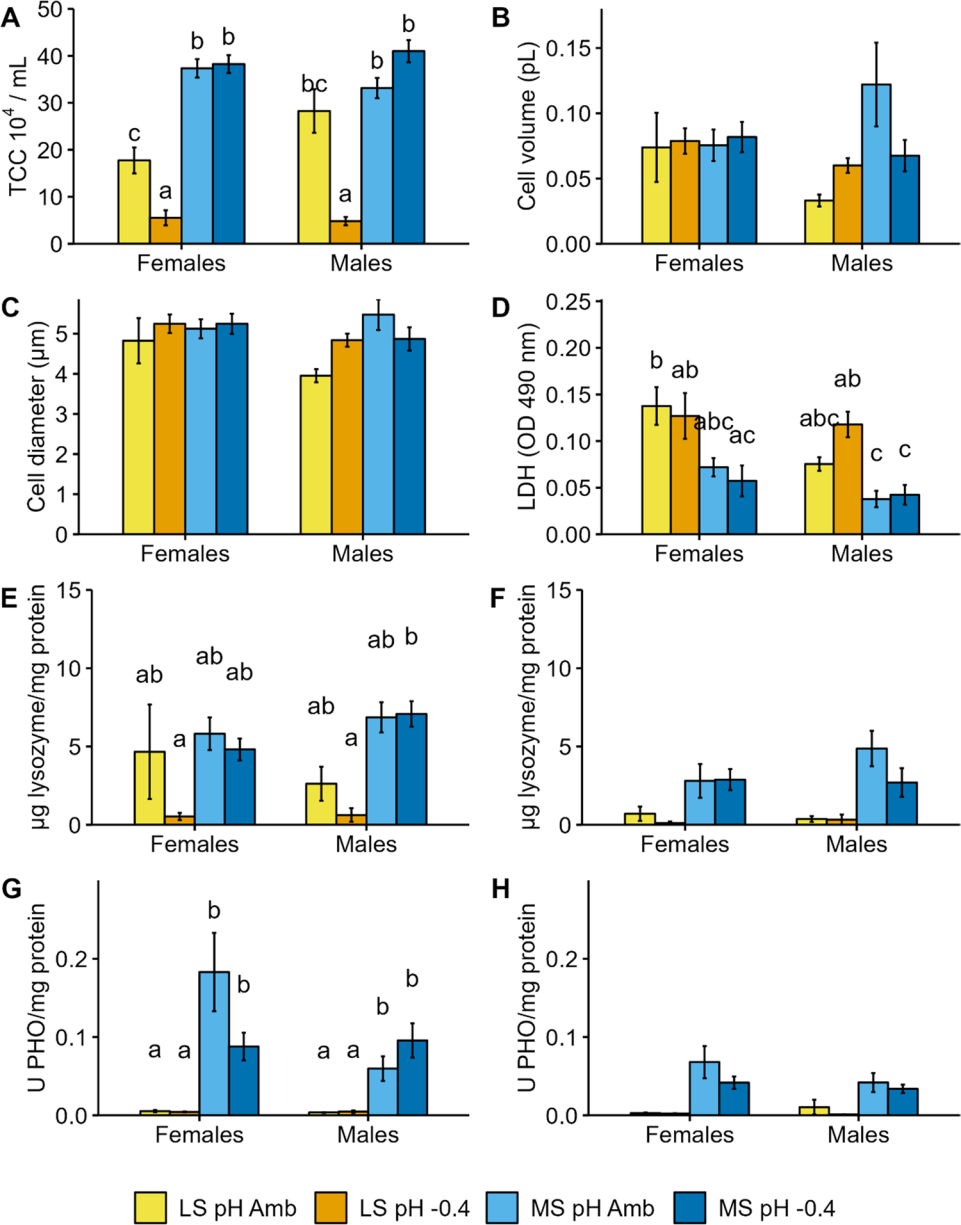
Results of immune biomarkers measured in whole coelomic fluid (**A** TCC, **B** cell volume, **C** cell diameter), coelomocytes (**D** LDH-cytotoxicity, **E** lysozyme activity, **G** acid phosphatase activity) or cell-free coelomic fluid (**F** lysozyme activity, **H** acid phosphatase activity) of sea urchins from lagoon site (LS) and marine site (MS) maintained for 8 months at ambient pH (pH Amb) and − 0.4 pH units (pH − 0.4). Different letters indicate significant differences among all conditions (*p* < 0.05)

**Fig. 3:**
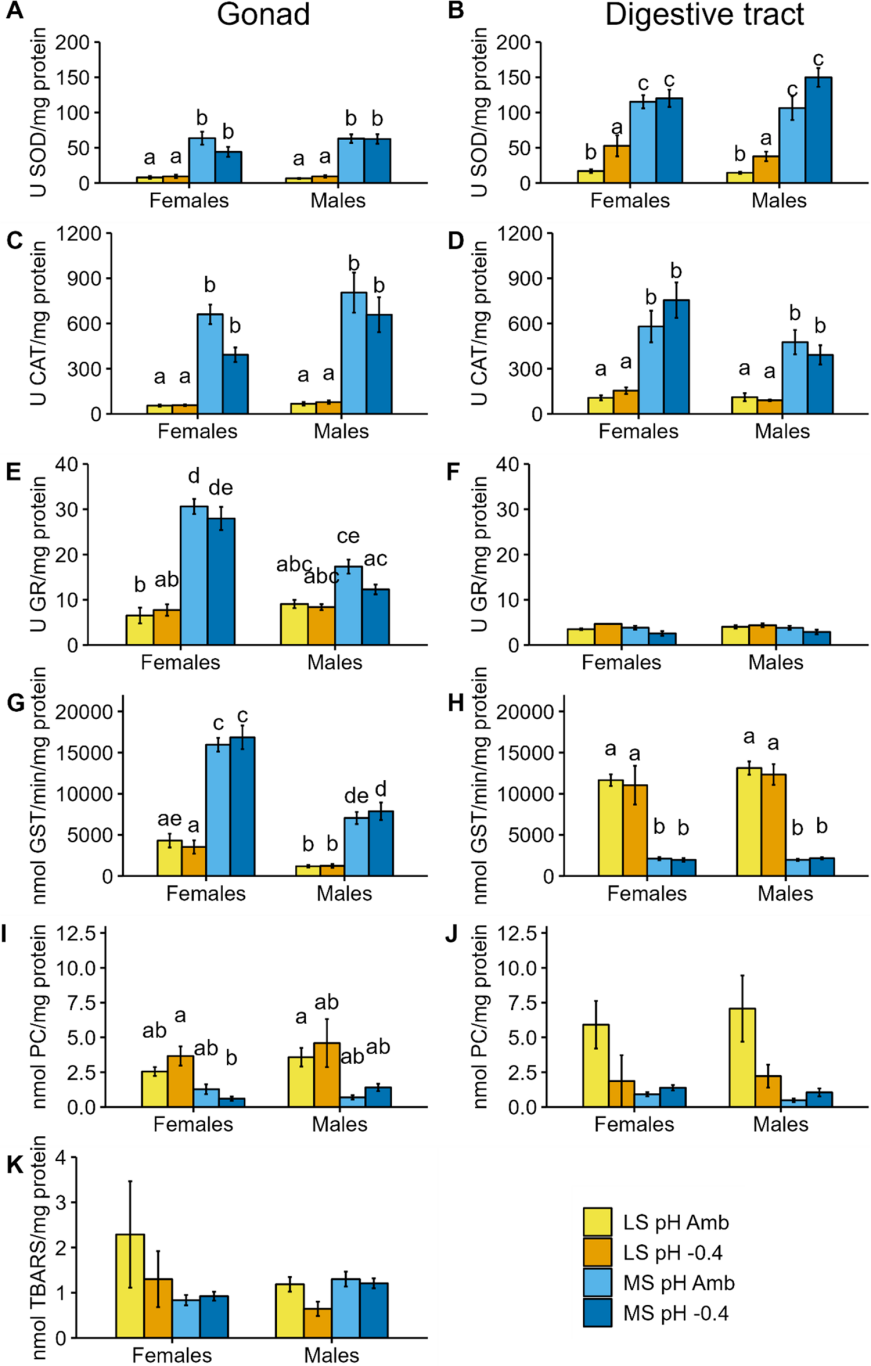
Results of biomarkers of antioxidant defence (**A**, **B**, **C**, **D**, **E**, **F**), xenobiotic metabolism (**G**, **H**), and oxidative damage (**I**, **J**, **K**) measured in gonad (**A** SOD activity, **C** CAT activity, **E** GR activity, **G** GST activity, **I** protein carbonyl content, **K** lipid peroxidation) and digestive tract (**B** SOD activity, **D** CAT activity, **F** GR activity, **H** GST activity, **J** protein carbonyl content) of sea urchins from lagoon site (LS) and marine site (MS) maintained for 8 months at ambient pH (pH Amb) and − 0.4 pH units (pH − 0.4). Mean ± standard error. Different letters indicate significant differences among all conditions (*p* < 0.05)

The PERMANOVA results highlighted a significant difference between the two sites, together with significant site:pH and site:pH:sex interactions (Table 2). The effects of the factors site, pH, sex, and their interactions, as assessed by mixed linear models for each biomarker, are shown in Tables 3 and 4 and Figs. 1, 2 and 3.

**Table 2:** Results of PERMANOVA performed on the complete dataset

Factor	Df	*F*. model	Partial *R*^2^	*p* value
Site	1	168.342	0.570	** < 0.001**
pH	1	5.462	0.019	0.111
Sex	1	1.342	0.005	0.239
Site:pH	1	39.427	0.133	** < 0.001**
Site:sex	1	2.585	0.009	0.168
pH:sex	1	0.005	0.000	0.963
Site:pH:sex	1	6.410	0.022	**0.029**
Residuals	72		0.244

**Table 3:** Mixed linear model results. The *p* values for all the immune biomarkers measured in the coelomic fluid of two groups of *P. lividus* from different sites maintained for 8 months at two pH levels (pH Amb and pH − 0.4) are reported. Significant results are in bold

	Coelomic fluid							
	TCC	Vol	Diam	LDH	Lys_C	Lys_F	PHO_C	PHO_F
Site	** < 0.001**	0.141	0.183	** < 0.001**	**0.002**	**0.024**	** < 0.001**	**0.008**
pH	** < 0.001**	0.178	0.104	0.570	**0.046**	0.556	0.975	0.536
Sex	0.325	0.122	0.060	**0.010**	0.541	0.924	0.447	0.167
Site:pH	** < 0.001**	0.060	0.079	0.482	0.133	0.816	0.867	0.420
Site:sex	0.094	0.226	0.280	0.301	0.170	0.857	0.785	0.592
pH:sex	0.647	0.684	0.849	**0.036**	0.184	0.920	0.203	0.342
Site:pH:sex	0.073	0.169	0.206	0.783	0.989	0.623	0.512	0.381

*TCC* total coelomocyte count, *Vol* coelomocyte volume, *Diam* coelomocyte diameter, *LDH* lactate dehydrogenase (cytotoxicity), *Lys* lysozyme, *PHO* acid phosphatase, *_C* coelomocytes, *_F* cell-free coelomic fluid

**Table 4:** Mixed linear model results. The *p* values for all the oxidative stress biomarkers measured in the gonads and digestive tract of *P. lividus* from different sites maintained for 8 months at two pH levels (pH Amb and pH − 0.4) are reported. Significant results are in bold

	Gonad						Digestive tract				
	SOD	CAT	GR	GST	PCC	LPO	SOD	CAT	GR	GST	PCC
Site	** < 0.001**	** < 0.001**	** < 0.001**	** < 0.001**	** < 0.001**	0.873	** < 0.001**	** < 0.001**	0.256	** < 0.001**	0.546
pH	0.938	0.395	0.734	0.893	0.604	0.058	**0.003**	0.288	0.755	0.719	0.526
Sex	0.063	**0.047**	**0.002**	** < 0.001**	0.169	**0.024**	0.844	**0.007**	0.166	0.361	0.259
Site:pH	0.193	0.082	0.073	0.397	0.508	0.078	**0.001**	0.994	0.070	0.518	**0.003**
Site:sex	0.320	0.941	** < 0.001**	0.156	0.519	**0.001**	0.441	0.459	0.573	0.507	**0.041**
pH:sex	0.449	0.407	0.200	0.456	0.109	0.179	0.267	0.194	0.601	0.263	0.257
Site:pH:sex	0.942	0.840	0.637	0.613	0.198	0.237	0.320	0.816	0.782	0.492	0.457

*SOD* superoxide dismutase, *CAT* catalase, *GR* glutathione reductase, *GST* glutathione-S-transferase, *PCC* protein carbonyl content, *LPO* lipid peroxidation

### Gonadal-somatic index

A significant difference between sites (*Χ*^2^ = 8.074, Df = 1; *p* = 0.005) and between sexes (*Χ*^2^ = 8.905, Df = 1; *p* = 0.003) was revealed by the linear mixed model. In particular, after 8 months of maintenance at the experimental conditions, MS animals had a higher GSI compared to LS, and females had always a higher GSI than males (Fig. 1). The post hoc test revealed a significant difference between MS females at pH − 0.4 and LS males at pH Amb (*p* = 0.010). No effects of pH or interactions between factors were highlighted.

### Immunological biomarkers

The differences in the various immune defence biomarkers analyzed in the two groups of sea urchins maintained for 8 months at two pH levels are mainly linked to the site of origin (Table 3). However, differences due to the other factors (i.e., pH and sex) were also present.

Significant effects of site (*Χ*^2^ = 161.527; Df = 1; *p* ≤ 0.001), pH (*Χ*^2^ = 20.052; Df = 1; *p* ≤ 0.001), and site:pH interaction (*Χ*^2^ = 54.156; Df = 1; *p* < 0.001) on TCC were found. TCC was higher in MS compared to LS animals. Within LS, sea urchins at low pH had the lowest TCC value (Fig. 2A), significantly different compared to the respective control condition. No significant effects of site, pH, or sex on coelomocyte volume and diameter were observed (Table 3; Fig. 2B–C).

Coelomocytes of sea urchins from MS showed significantly lower cytotoxicity levels (LDH; Fig. 2D) compared to those from LS animals (Χ^2^ = 33.021; Df = 1; *p* < 0.001). Differences were found also between sexes (*Χ*^2^ = 6.622; Df = 1; *p* = 0.010). Interestingly, the response in the two sexes varied with the pH of exposure (pH:sex interaction; *Χ*^2^ = 4.402; Df = 1; *p* = 0.036), with higher values for females at pH Amb and for males at pH − 0.4, even though the Tukey post hoc test did not reveal significant differences within sea urchins from the same site.

The coelomocyte (*Χ*^2^ = 9.873; Df = 1; *p* = 0.002) and CFCF (*Χ*^2^ = 5.060; Df = 1; *p* = 0.025) lysozyme activities were significantly different between the two groups of sea urchins (Fig. 2E–F). In both LS and MS animals, pH significantly affected the enzyme activity in coelomocytes (*Χ*^2^ = 3.989; Df = 1; *p* = 0.046), but not in CFCF (*Χ*^2^ = 0.347; Df = 1; *p* = 0.556). Furthermore, influence of animal sex and the interaction among factors were not significant (Table 3).

Lastly, the acid phosphatase activity was significantly different between sites in both coelomocytes (*Χ*^2^ = 50.542; Df = 1; *p* < 0.001) and CFCF (*Χ*^2^ = 7.067; Df = 1; *p* = 0.008). Enzymatic activity was significantly lower in LS compared to MS animals (Fig. 2G–H). No differences due to pH or sex or the interactions of the factors were highlighted (Table 3).

### Oxidative stress biomarkers

The oxidative stress biomarkers analyzed in the two groups of sea urchins maintained for 8 months at two pH levels showed a clear difference between sites of origin and sexes (Table 4). A significant influence of the exposure pH was limited to SOD activity analyzed in the digestive tract and the interaction site:pH was significant only for SOD and PCC in the digestive tract.

SOD activity (Fig. 3A–B) was significantly lower in both gonads (*Χ*^2^ = 115.711; Df = 1; *p* < 0.001) and digestive tract (*Χ*^2^ = 225.680; Df = 1; *p* < 0.001) of LS sea urchins. Significant effects of pH (*Χ*^2^ = 21.661; Df = 1; *p* < 0.001) and the site:pH interaction (*Χ*^2^ = 11.126; Df = 1; *p* < 0.001) were highlighted for the SOD activity in the digestive tract. On average, SOD activity was slightly higher in gonadal tissue of males compared to females, but not significantly (*Χ*^2^ = 3.450; Df = 1; *p* = 0.063).

CAT activity (Fig. 3C–D) of *P. lividus* was significantly higher in MS compared to LS in both gonads (*Χ*^2^ = 166.288; Df = 1; *p* < 0.001) and digestive tract (*Χ*^2^ = 97.239; Df = 1; *p* < 0.001). Moreover, in both tissues, significant differences between sexes were highlighted in the l mm (gonad, *Χ*^2^ = 3.946; Df = 1; *p* = 0.047; digestive tract, *Χ*^2^ = 7.168; Df = 1; *p* = 0.007), but not in the post hoc test. In particular, higher values were observed in the gonadal tissue of males and the digestive tract of females.

The activity of GR in the gonads (Fig. 3E) of the experimental sea urchins was different based on the factors site (*Χ*^2^ = 67.722; Df = 1; *p* < 0.001), sex (*Χ*^2^ = 9.197; Df = 1; *p* = 0.002) and the interaction site:sex (*Χ*^2^ = 28.633; Df = 1; *p* < 0.001). Enzymatic activity was always higher in MS animals. In LS, males had the higher U GR/mg protein levels, whereas in MS males had the lowest. No effects of pH were revealed in *P. lividus* gonads. Moreover, no differences were observed in the GR activity in the digestive tract (Fig. 3F, Table 4).

GST activity showed an opposite pattern between measurements carried out in gonads and digestive tract (Fig. 3G–H). For both tissues a significant difference between sites (gonad, *Χ*^2^ = 269.481; Df = 1; *p* < 0.001; digestive tract, *Χ*^2^ = 607.294; Df = 1; *p* < 0.001) was found. However, higher values of GR activity were measured in the gonad for MS, and in the digestive tract for LS animals. Besides, in the gonads, a significant influence of sex was observed (*Χ*^2^ = 109.382; Df = 1; *p* < 0.001), with males showing lower values compared to females in both sites (Fig. 3G). No differences due to pH levels were observed either in the gonad or in the digestive tract (Table 4).

Oxidative damage (PCC, Fig. 3I–J and LPO, Fig. 3K) was higher in LS animals. However, the difference between sites was significant only for PCC measured in the gonads (*Χ*^2^ = 14.315; Df = 1; *p* < 0.001). In the digestive tract, PCC was significantly influenced by the interactions site:pH (*Χ*^2^ = 8.551; Df = 1; *p* = 0.004) and site:sex (*Χ*^2^ = 4.172; Df = 1; *p* = 0.041). Indeed, PCC values were higher at pH Amb compared to pH − 0.4 in LS animals, whereas the opposite situation was found in MS ones (Fig. 3J).

Statistical analyses highlighted significant differences in the gonad LPO between sexes (*Χ*^2^ = 5.117; Df = 1; *p* = 0.024), but with a different response of sexes between sites (significant site:sex interaction; *Χ*^2^ = 11.453; Df = 1; *p* < 0.001). Indeed, females showed higher values in LS sea urchins, whereas males showed higher values in MS ones (Fig. 3K). Results showed differences due to pH levels close to significance (*p* = 0.058, Table 4), but they are visible only in LS animals (Fig. 3K).

### Canonical correlation analysis

**Fig. 4:**
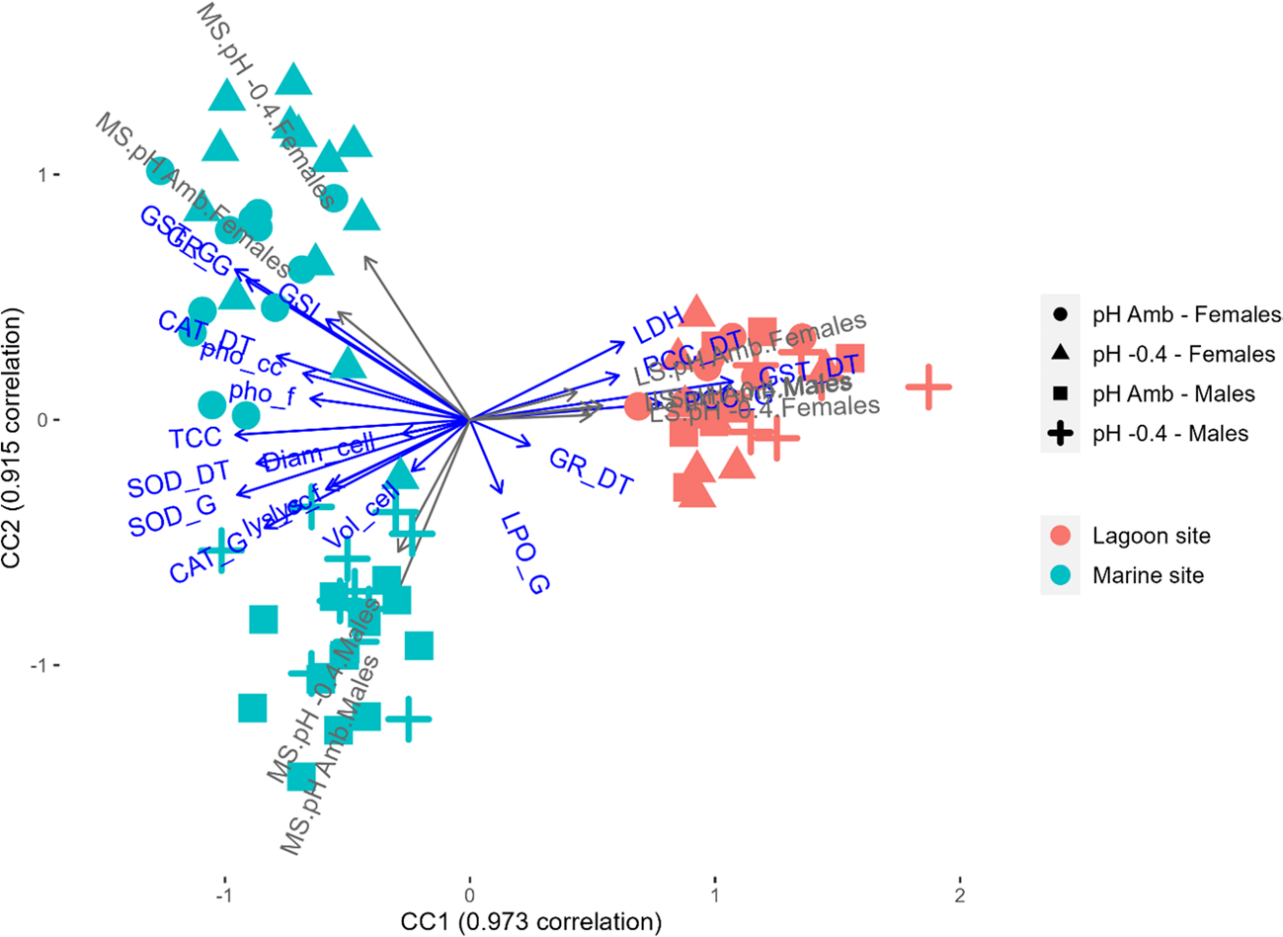
Canonical correlation analysis (CCA) plot for the biomarker responses in *P. lividus* collected in two sites, exposed to different pH levels (pH Amb and pH − 0.4). GSI, gonadal-somatic index; TCC, total coelomocyte count; Vol_cell, coelomocyte volume; Diam_cell, coelomocyte diameter; lys, lysozyme; pho, acid phosphatase activity; LDH, lactate dehydrogenase; SOD, superoxide dismutase activity; CAT, catalase activity; GR, glutathione reductase activity; GST, glutathione-S-transferase activity; PCC, protein carbonyl content; LPO, lipid peroxidation; _cc, coelomocytes; _f, cell-free coelomic fluid; _G, gonads; _DT, digestive tract

In Fig. 4, the biplot showed the first two components of the CCA analysis. The explained correlations were 97.3% and 91.5% for the first and the second canonical correlation, respectively.

The two sites were well separated along the CC1, with MS on the left and LS on the right of the plot. Sea urchins from LS were characterized by higher activity levels of the enzymes LDH, GR, and GST in the digestive tract and high levels of oxidative damage in terms of PCC in both tissues. Instead, MS animals were identified by greater GSI and higher levels of all the other biomarkers, except for LPO levels. Within LS, a clear separation between sexes was not visible. Differently, females and males from MS had an opposite position along the CC2, which appeared to be influenced by higher LPO levels for males. Lastly, a clear separation between pH levels was not present (Fig. 4).

## Discussion

Changes in seawater chemistry due to CO_2_ uptake and related acidification processes pose serious threats to marine life. In sea urchins, biological characteristics such as calcification, physiology, behavior, and reproduction can be modified and disrupted by reduced pH (Asnicar and Marin 2022; Dupont et al. 2013; Gallo et al. 2020). However, adults of some species of sea urchin (e.g., *P. lividus*, *Stomopneustes variolaris*, *Echinometra* sp., *Sterechinus neumayeri*) showed acclimation capability under long-term exposure to seawater acidification (Asnicar et al. 2021; Shetye et al. 2020; Hazan et al. 2014; Uthicke et al. 2020; Morley et al. 2016).

Many questions are still unanswered, however, especially regarding the impact on the immunological and antioxidant status after long-term maintenance at reduced pH, or the variability in the responses among local populations of the same species. In this perspective, we maintained two groups of the sea urchin *P. lividus* for 8 months to reduced pH (− 0.4 units compared to ambient) to assess their gonadal growth, as well as a battery of 12 immunological, oxidative stress, and detoxification biomarkers.

In the companion paper by Asnicar et al. (2021), after being maintained for 6 months at − 0.4 pH units compared to ambient, these *P. lividus* specimens showed acclimation capability in physiological and behavioral responses. In line with those findings, in the present study, after 8 months of exposure, sea urchins showed very limited differences between pH levels in the investigated biomarkers.

GSI, although higher in animals maintained at reduced pH, was not significantly different between pH conditions. This is in accordance with other long-term experiments carried out with pH scenarios expected by the end of the present century (reduction of 0.3–0.4 pH units IPCC RCP 8.5). Indeed, the GSI was not impaired in *S. neumayeri* (Dell’Acqua et al. 2019), *T. gratilla* (Dworjanyn and Byrne 2018), *Strongylocentrotus fragilis* (Taylor et al. 2014), *Echinometra* sp. (Hazan et al. 2014), as well as in *P. lividus* (Marčeta et al. 2020). However, more severe pH reduction conditions, which may be reached beyond 2100, had a negative impact on the GSI of sea urchins (Dworjanyn and Byrne 2018; Taylor et al. 2014).

After 8 months of exposure, the coelomocyte characteristics and functions investigated in the present study were slightly affected by the pH reduction. Indeed, in LS sea urchins, the TCC and the lysozyme activity in coelomocytes were significantly lower in the low pH condition. Conversely, MS animals did not show significant alterations in their immunological variables. In a recent study, some of the biomarkers analyzed here were measured in *P. lividus* from the lagoon of Venice after a 2-month exposure at pH 8.0, 7.7, and 7.4 (Marčeta et al. 2020). Similar to our findings, the TCC and lysozyme activity in coelomocytes were significantly altered in low pH conditions, more markedly at pH 7.4 than at 7.7. Comparing ours to Marčeta et al. results, at pH 7.7, the variation patterns of the two immunomarkers were different between 2 and 8 months of exposure, indicating a time-dependent modulation of the response.

In *P. lividus* sampled within a CO_2_-vent system, no differences were found in TCC compared to a control site (Migliaccio et al. 2019). However, the observed changes in the proteomic profile suggested enhancement of the immune defence system which stimulates protective mechanisms promoting adaptation to stress conditions (Migliaccio et al. 2019).

In the present study, values of the immunological biomarkers investigated were lower compared to those obtained in a shorter exposure by Marčeta et al. (2020) and to those from animals collected in a CO_2_-vent system (Migliaccio et al. 2019). This may indicate that the sea urchins were in an immunoquiescent state (Alijagic et al. 2021; Gross et al. 1999), suggesting that acclimation to low pH occurred. Indeed, animals did not need to face other environmental stressors as they were maintained in aquaria without any significant disturbance. Unlike MS animals, in LS sea urchins the very low values of TCC at pH − 0.4 may be linked to the differential energetic investment in favor of gonadal or somatic growth. Further studies should be carried out to better understand the mechanism of energetic investment throughout gametogenesis in animals maintained at low pH.

The increase in *p*CO_2_ and H^+^ concentration in seawater and the derived hypercapnia can affect acid–base homeostasis and metabolic functions leading to oxidative stress (Pörtner et al. 2004, 2005). Lewis et al. (2016) found that by modulating antioxidant enzymatic activity and bicarbonate concentration, the coelomic fluid pH and oxidative damage were maintained similar to control conditions. Comparably, *P. lividus* specimens sampled within a CO_2_-vent system presented higher total antioxidant capacity levels compared to control from non-vent areas and, as a result, the oxidative damage did not differ between sampling sites (Migliaccio et al. 2019). Likewise, in the present work, the antioxidant enzymes were slightly modulated by the pH of exposure. Although oxidative damage was not significantly influenced by pH, PCC in the digestive tract showed lower values in sea urchins maintained under low pH. Possibly, in those animals, the slightly higher levels of SOD and CAT led to better protection from oxidative damage. In particular, SOD activity was significantly higher in the digestive tract of sea urchins maintained at low pH. Similarly, in *P. lividus*, Marčeta et al. (2020) found that at low pH, the digestive tract is the tissue most affected by oxidative stress, showing significantly increased CAT activity compared to control conditions. Overall, *P. lividus* antioxidant capacity showed to be resilient to seawater acidification conditions also in previous experiments. However, more severe pH conditions or sampling in different gametogenic stages might highlight a greater impact on the immunological and antioxidant response in sea urchins. Indeed, after a spawning event, immune and antioxidant systems can be compromised, resulting in higher susceptibility to environmental stressors (Fernández-Boo et al. 2018; Ghobeishavi et al. 2016; Kim et al. 2022).

Compared to the results obtained after 2 months of exposure by Marčeta et al. (2020), the values of SOD and CAT activities measured in the present work were considerably higher. It is known that during the peak of gametogenesis, sea urchins maintain higher antioxidant levels (Amri et al. 2017). Increased antioxidant enzyme activities can therefore be justified considering that GSI values in the present study were 4–5 times higher than those reported by Marčeta et al. (2020). In our results, on the other hand, there is a great difference between sexes.

Significant dissimilarities between sexes were found in the GSI, with males having a lower GSI than females, in the coelomocyte cytotoxicity, with males showing lower levels, and in several oxidative stress biomarkers (CAT, GR, GST, and LPO in the gonads; CAT in the digestive tract).

Most of the dissimilarities found between sexes involved oxidative stress biomarkers in the gonad. CAT activity was higher in males, whereas GR and GST activities and LPO levels were higher in females. In *P. lividus*, the two sexes showed inherent differences in the gonad, for example, in the number of genes involved in fatty acid metabolism, membrane phospholipid production, and sex determination (Machado et al. 2022). Some other understudied genes involved in antioxidant processes may be differentially expressed in the gonadal tissue and would explain the differences found in the present study.

Similar to our results, Arizza et al. (2013) found that cytotoxicity (as LDH activity) was higher in females than in males.

Studies demonstrated that immune and antioxidant systems in invertebrate females are more active than in males, to increase the protection of eggs (Arizza et al. 2013; Wu and Chu 2010). In the context of seawater acidification, recent studies suggested that the maternal contribution to the success of the next generation is more relevant than the paternal (Palombo et al. 2023; Munari et al. 2022). This hypothesis might explain why antioxidant biomarker levels in our study were higher in females. Indeed, sea urchins were in the growing phase of the gametogenesis, when an increased level of defence is useful to ensure good offspring quality.

However, none of the pH:Sex interactions were significant (with the exception of LDH). Marčeta et al. (2020) found, instead, that the differences in SOD and CAT activity among pH levels were significantly more pronounced in females than in males. Dissimilarities between the studies might be related to the gametogenic phase of the sea urchins with broader differences between sexes at an earlier maturation stage.

Differences between sexes are more pronounced in MS than in LS animals. As shown in the CCA plot, LS values are more similar among them compared to MS values, which are widely spread on the plot area. LS individuals’ responses overlap without a clear distinction between sexes or pH levels. On the other hand, MS specimens are distinctly separated by animals’ sex.

The CCA plot sheds light on another important separation that is statistically significant in almost all the endpoints investigated (15 out of 20). Among the three factors considered in the statistical analyses (i.e., site, pH, and sex), the clearest effect is certainly due to the animals’ site of origin. GSI, as well as immunological and oxidative stress biomarkers, revealed wide and significant differences between the two sites. As shown by the PERMANOVA analysis, animals from different sites responded differently to the pH (significant site:pH interaction). Indeed, although not clearly separated, clusters of pH levels are visible for MS animals. Overall, the variability of the sea urchins’ responses to low pH is greater in animals from MS compared to LS.

It has been proven that sea urchins within the Adriatic Sea (where the sampling sites are located) belong to the same genetic population (Paterno et al. 2017). Hence, the differences highlighted between sites are not genetically driven.

Local biological adaptation is a crucial point to consider when investigating seawater acidification effects (Vargas et al. 2017, 2022). Differences depending on the sampling area have been documented for immunological and antioxidant variables in previous studies. For example, the coelomocyte number was different in *P. lividus* from two sites in the Gulf of Naples (Migliaccio et al. 2019). During the first 6 months of exposure, LS and MS sea urchins maintained at − 0.4 pH units showed different duration in the period needed for acclimation to reduced pH, as revealed by their physiological and behavioral responses (Asnicar et al. 2021). As shown in the previous work (Asnicar et al. 2021), LS animals acclimated much faster to seawater acidification than MS ones. This is possibly related to the ecological characteristics of the sampling sites. LS is located in the Lagoon of Venice which is characterized by high daily and seasonal fluctuations in environmental parameters (temperature, salinity, dissolved oxygen, chlorophyll content, pH, sediment resuspension) and high anthropogenic disturbance (pollution, marine traffic, noise). MS is located in a more stable environment, far from remarkable freshwater input and high human direct impact (see Supplementary Material and Asnicar et al. 2021 for details). Fluctuations of environmental parameters may play a role in the sensitivity and resilience of sea urchins and other marine species to experimental challenges (Bednaršek et al. 2021; Vargas et al. 2022). At some locations, animals might be already preadapted to future scenarios of global change as they live in highly variable environments where pH values can change drastically throughout the day or season. Therefore, comparisons of the responses to environmental stressors in different local populations are crucial to understanding the extent of potential impacts on biodiversity and species distribution. In this context, it is important to notice that, although seawater parameters measurements in the two collection sites are limited, pH values as low as the tested ones (LS, pH = 7.61 ± 0.12; MS = 7.64 ± 0.07; average ± standard deviation) have not been measured in the two collection sites LS and MS (Table S1, Figure S1). It is reasonable to assume that both sea urchin groups never experienced a pH as low as the tested one throughout their lifetime upon collection.

In conclusion, the present study showed significant differences in immunological and antioxidant responses of sea urchins from different environments, when subject to reduced pH. Sex-specific differences were observed in the GSI, as well as in the modulation of immunological responses, and antioxidant status. Generally, females exhibited higher values in these parameters. However, the impact of pH exposure on sex differences was limited. Like many other sea urchins, *P. lividus* is a keystone species in coastal environments where depletion of its populations can cause profound changes in the ecosystem. It is also a commercially important resource exploited for human consumption. Increased harvesting and global change–related pressures are threatening the maintenance of wild populations. In this context, improved knowledge of the biological performances of *P. lividus* under different environmental stressors can promote species conservation and aquaculture practices. To the best of our knowledge, immunological and antioxidant defence biomarkers have been very scarcely analyzed in *P. lividus* and other sea urchins exposed to seawater acidification, and generally they were measured after shorter exposures. Our results demonstrate that long-term exposure is not enough to completely exclude the persistence of oxidative or immunological stress, but the impact of environmental stressors may differ in sea urchins from different populations or different locations.

Further studies assessing the co-occurrence of other environmental stressors are needed to fully understand the implications for coastal management and for broodstock collection in sea urchin aquaculture.

## Supplementary Information

Below is the link to the electronic supplementary material.

Supplementary file1 (DOCX 751 KB)

## Data Availability

The data that support the findings of this study are available from the corresponding author upon reasonable request.

## References

[CR1] Aebi H (1984) Catalase in vitro, in: Methods in enzymology. Academic Press, pp. 121–126. 10.1016/S0076-6879(84)05016-3 10.1016/s0076-6879(84)05016-3 6727660

[CR2] Alijagic A, Bonura A, Barbero F, Puntes VF, Gervasi F, Pinsino A (2021) Immunomodulatory function of polyvinylpyrrolidone (PVP)-functionalized gold nanoparticles in *Vibrio*-stimulated sea urchin immune cells. Nanomaterials 11:2646. 10.3390/nano11102646 34685085 10.3390/nano11102646 PMC8539316

[CR3] Amri S, Samar MF, Sellem F, Ouali K (2017) Seasonal antioxidant responses in the sea urchin *Paracentrotus lividus* (Lamarck 1816) used as a bioindicator of the environmental contamination in the South-East Mediterranean. Mar Pollut Bull 122:392–402. 10.1016/j.marpolbul.2017.06.079 28705630 10.1016/j.marpolbul.2017.06.079

[CR4] Anand M, Rangesh K, Maruthupandy M, Jayanthi G, Rajeswari B, Priya RJ (2021) Effect of CO_2_ driven ocean acidification on calcification, physiology and ovarian cells of tropical sea urchin *Salmacis virgulata* – a microcosm approach. Heliyon 7:e05970. 10.1016/j.heliyon.2021.e05970 33521355 10.1016/j.heliyon.2021.e05970 PMC7820546

[CR5] Arizza V, Vazzana M, Schillaci D, Russo D, Giaramita FT, Parrinello N (2013) Gender differences in the immune system activities of sea urchin *Paracentrotus lividus*. Comp Biochem Physiol - Mol Integr Physiol. 10.1016/j.cbpa.2012.11.021 10.1016/j.cbpa.2012.11.021 23220062

[CR6] Asnicar D, Marin MG (2022) Effects of seawater acidification on echinoid adult stage: a review. J Mar Sci Eng 10:477. 10.3390/jmse10040477 10.3390/jmse10040477

[CR7] Asnicar D, Zanovello L, Badocco D, Munari M, Marin MG (2022) Different ecological histories of sea urchins acclimated to reduced pH influence offspring response to multiple stressors. Environ Res 212:113131. 10.1016/j.envres.2022.113131 35337831 10.1016/j.envres.2022.113131

[CR8] Asnicar D, Novoa-Abelleira A, Minichino R, Badocco D, Pastore P, Finos L, Munari M, Marin MG (2021) When site matters: metabolic and behavioural responses of adult sea urchins from different environments during long-term exposure to seawater acidification. Mar Environ Res: 105372. 10.1016/j.marenvres.2021.105372 10.1016/j.marenvres.2021.105372 34058626

[CR9] Badocco D, Pedrini F, Pastore A, di Marco V, Marin MG, Bogialli S, Roverso M, Pastore P (2021) Use of a simple empirical model for the accurate conversion of the seawater pH value measured with NIST calibration into seawater pH scales. Talanta 225:122051. 10.1016/j.talanta.2020.122051 33592773 10.1016/j.talanta.2020.122051

[CR10] Bednaršek N, Calosi P, Feely RA, Ambrose R, Byrne M, Chan KYK, Dupont S, Padilla-Gamiño JL, Spicer JI, Kessouri F, Roethler M, Sutula M, Weisberg SB (2021) Synthesis of thresholds of ocean acidification impacts on echinoderms. Front Mar Sci 8:261. 10.3389/fmars.2021.602601 10.3389/fmars.2021.602601

[CR11] Bisanti L, La Corte C, Dara M, Bertini F, Parrinello D, Chemello R, Cammarata M, Parisi MG (2024) How does warmer sea water change the sensitivity of a Mediterranean thermophilic coral after immune-stimulation? Coral Reefs 43:137–150. 10.1007/s00338-023-02454-9 10.1007/s00338-023-02454-9

[CR12] Boudouresque CF, Verlaque M (2020) *Paracentrotus lividus*, In: Lawrence JM (Ed.) Sea urchins: Biology and Ecology. Elsevier B.V., pp. 447–485. 10.1016/B978-0-12-819570-3.00026-3

[CR13] Bradford MM (1976) A rapid and sensitive method for the quantitation of microgram quantities of protein utilizing the principle of protein-dye binding. Anal Biochem 72:248–254 942051 10.1016/0003-2697(76)90527-3

[CR14] Bressan M, Chinellato A, Munari M, Matozzo V, Manci A, Marčeta T, Finos L, Moro I, Pastore P, Badocco D, Marin MG (2014) Does seawater acidification affect survival, growth and shell integrity in bivalve juveniles? Mar Environ Res 99:136–148. 10.1016/j.marenvres.2014.04.009 24836120 10.1016/j.marenvres.2014.04.009

[CR15] Buege JA, Aust SD (1978) Microsomal lipid peroxidation. In: Fleischer S, Packer LBTM (eds) Methods in enzymology. Academic Press, pp 302–310. 10.1016/S0076-6879(78)52032-6 10.1016/s0076-6879(78)52032-6 672633

[CR16] Byrne M, Ho M, Wong E, Soars NA, Selvakumaraswamy P, Shepard-Brennand H, Dworjanyn SA, Davis AR (2011) Unshelled abalone and corrupted urchins: development of marine calcifiers in a changing ocean. Proc R Soc B: Biol Sci 278:2376–2383. 10.1098/rspb.2010.2404 10.1098/rspb.2010.2404 PMC3119014 21177689

[CR17] Calosi P, Melatunan S, Turner LM, Artioli Y, Davidson RL, Byrne JJ, Viant MR, Widdicombe S, Rundle SD (2017) Regional adaptation defines sensitivity to future ocean acidification. Nat Commun 8:13994. 10.1038/ncomms13994 28067268 10.1038/ncomms13994 PMC5227702

[CR18] Cao Z, Mu F, Wei X, Sun Y (2015) Influence of CO_2_-induced seawater acidification on the development and lifetime reproduction of *Tigriopus japonicus* Mori, 1938. J Nat Hist 49:2813–2826. 10.1080/00222933.2015.1034213 10.1080/00222933.2015.1034213

[CR19] Castillo N, Saavedra LM, Vargas CA, Gallardo-Escárate C, Détrée C (2017) Ocean acidification and pathogen exposure modulate the immune response of the edible mussel *Mytilus chilensis*. Fish Shellfish Immunol 70:149–155. 10.1016/j.fsi.2017.08.047 28870859 10.1016/j.fsi.2017.08.047

[CR20] Conradi M, Sánchez-Moyano JE, Bhuiyan MKA, Rodríguez-Romero A, Galotti A, Basallote MD, DelValls A, Parra G, Riba I (2019) Intraspecific variation in the response of the estuarine European isopod *Cyathura carinata* (Krøyer, 1847) to ocean acidification. Sci Total Environ 683:134–145. 10.1016/j.scitotenv.2019.05.227 10.1016/j.scitotenv.2019.05.227 31129324

[CR21] Crapo JD, McCord JM, Fridovich I (1978) Preparation and assay of superoxide dismutases, in: Methods in Enzymology. pp. 382–393. 10.1016/S0076-6879(78)53044-9 10.1016/s0076-6879(78)53044-9 362127

[CR22] Csallany AS, Guan MD, Manwaring JD, Addis PB (1984) Free malonaldehyde determination in tissues by high-performance liquid chromatography. Anal Biochem 142:277–283. 10.1016/0003-2697(84)90465-2 6528969 10.1016/0003-2697(84)90465-2

[CR23] Dalle-Donne I, Rossi R, Giustarini D, Milzani A, Colombo R (2003) Protein carbonyl groups as biomarkers of oxidative stress. Clin Chim Acta 329(1–2):23–38. 10.1016/S0009-8981(03)00003-2 12589963 10.1016/S0009-8981(03)00003-2

[CR24] Damiens G, Gnassia-Barelli M, Loquès F, Roméo M, Salbert V (2007) Integrated biomarker response index as a useful tool for environmental assessment evaluated using transplanted mussels. Chemosphere 66:574–583. 10.1016/j.chemosphere.2006.05.032 16828146 10.1016/j.chemosphere.2006.05.032

[CR25] Dell’Acqua O, Ferrando S, Chiantore M, Asnaghi V (2019) The impact of ocean acidification on the gonads of three key Antarctic benthic macroinvertebrates. Aquat Toxicol 210:19–29. 10.1016/j.aquatox.2019.02.012 30818112 10.1016/j.aquatox.2019.02.012

[CR26] Doney SC, Fabry VJ, Feely RA, Kleypas JA (2009) Ocean acidification: the other CO_2_ problem. Ann Rev Mar Sci 1:169–192. 10.1146/annurev.marine.010908.163834 10.1146/annurev.marine.010908.163834 21141034

[CR27] Dupont S, Thorndyke M (2012) Relationship between CO_2_-driven changes in extracellular acid-base balance and cellular immune response in two polar echinoderm species. J Exp Mar Biol Ecol 424–425:32–37. 10.1016/j.jembe.2012.05.007 10.1016/j.jembe.2012.05.007

[CR28] Dupont S, Dorey N, Stumpp M, Melzner F, Thorndyke M (2013) Long-term and trans-life-cycle effects of exposure to ocean acidification in the green sea urchin *Strongylocentrotus droebachiensis*. Mar Biol 160:1835–1843. 10.1007/s00227-012-1921-x 10.1007/s00227-012-1921-x

[CR29] Dworjanyn SA, Byrne M (2018) Impacts of ocean acidification on sea urchin growth across the juvenile to mature adult life-stage transition is mitigated by warming. Proc R Soc B: Biol Sci. 10.1098/rspb.2017.2684 10.1098/rspb.2017.2684 PMC5904309 29643209

[CR30] Endean R (1966) The coelomocytes and coelomic fluids. In: Boolootian RA (ed) Physiology of Echinodermata. Interscience Publishers, New York, pp 301–328

[CR31] FAO (2021) FAO’s work on climate change - Fisheries and aquaculture 2020. FAO. 10.4060/cb3414en 10.4060/cb3414en

[CR32] Fernández-Boo S, Pedrosa-Oliveira MH, Afonso A, Arenas F, Rocha F, Valente LMP, Costas B (2018) Annual assessment of the sea urchin (*Paracentrotus lividus*) humoral innate immune status: tales from the north Portuguese coast. Mar Environ Res 141:128–137. 10.1016/j.marenvres.2018.08.007 30139531 10.1016/j.marenvres.2018.08.007

[CR33] Finos L (2020) r41sqrt10: Livio’s Sandbox. R Package Version 0.1. https://github.com/livioivil/r41sqrt10/. Accessed 30 Jan 2024

[CR34] Fransner F, Fröb F, Tjiputra J, Goris N, Lauvset SK, Skjelvan I, Jeansson E, Omar A, Chierici M, Jones E, Fransson A, Ólafsdóttir SR, Johannessen T, Olsen A (2022) Acidification of the Nordic seas. Biogeosciences 19:979–1012. 10.5194/bg-19-979-2022 10.5194/bg-19-979-2022

[CR35] Gallo A, Esposito MC, Cuccaro A, Buia MC, Tarallo A, Monfrecola V, Tosti E, Boni R (2020) Adult exposure to acidified seawater influences sperm physiology in *Mytilus galloprovincialis*: laboratory and in situ transplant experiments. Environ Pollut 265:115063. 10.1016/j.envpol.2020.115063 32806401 10.1016/j.envpol.2020.115063

[CR36] Gattuso JP, Hansson L (2011) Ocean acidification. Oxford University Press, p 352

[CR37] Ghobeishavi A, Mousavi SM, Yavari V, Kochanian P, Zakeri M (2016) The innate immunity changes of the female anadromous hilsa shad, *Tenualosa ilisha*, during spawning and post spawning season. Iran J Fish Sci 15(4):1526–1539. 10.22092/ijfs.2018.114627 10.22092/ijfs.2018.114627

[CR38] Grosjean P, Spirlet C, Gosselin P, Vaïtilingon D, Jangoux M (1998) Land-based, closed-cycle echiniculture of *Paracentrotus lividus* (Lamarck) (Echinoidea: Echinodermata): a long-term experiment at a pilot scale. J Shellfish Res 17:1523–1531

[CR39] Gross PS, Al-Sharif WZ, Clow LA, Smith LC (1999) Echinoderm immunity and the evolution of the complement system. Dev Comp Immunol 23:429–442. 10.1016/S0145-305X(99)00022-1 10426433 10.1016/S0145-305X(99)00022-1

[CR40] Habig WH, Pabst MJ, Jakoby WB (1974) Glutathione S-transferases. J Biol Chem 249:7130–7139. 10.1016/S0021-9258(19)42083-8 4436300 10.1016/S0021-9258(19)42083-8

[CR41] Hazan Y, Wangensteen OS, Fine M (2014) Tough as a rock-boring urchin: adult *Echinometra* sp. EE from the Red Sea show high resistance to ocean acidification over long-term exposures. Mar Biol 161:2531–2545. 10.1007/s00227-014-2525-4 10.1007/s00227-014-2525-4

[CR42] Holtmann WC, Stumpp M, Gutowska MA, Syré S, Himmerkus N, Melzner F, Bleich M (2013) Maintenance of coelomic fluid pH in sea urchins exposed to elevated CO_2_: the role of body cavity epithelia and stereom dissolution. Mar Biol 160:2631–2645. 10.1007/s00227-013-2257-x 10.1007/s00227-013-2257-x

[CR43] Hoshijima U, Hofmann GE (2019) The variability of seawater chemistry in a kelp forest environment is linked to transgenerational effects in situ in the purple sea urchin, *Strongylocentrotus purpuratus*. Front Mar Sci 6:62. 10.3389/fmars.2019.00062 10.3389/fmars.2019.00062

[CR44] IPCC (2014) Climate change 2014: synthesis report. contribution of working groups i, ii and iii to the fifth assessment report of the intergovernmental panel on climate change. [Core Writing Team, R.K. Pachauri and L.A. Meyer (eds) ] IPCC, Geneva, Switzerland, pp 151. https://www.ipcc.ch/site/assets/uploads/2018/05/SYR_AR5_FINAL_full_wcover.pdf

[CR45] IPCC (2019) IPCC Special Report on the Ocean and Cryosphere in a Changing Climate. [H.-O. Pörtner, D.C. Roberts, V. Masson-Delmotte, P. Zhai, M. Tignor, E. Poloczanska, K. Mintenbeck, A. Alegría, M. Nicolai, A. Okem, J. Petzold, B. Rama, N.M. Weyer (eds) ] pp 755. https://www.ipcc.ch/site/assets/uploads/sites/3/2019/12/SROCC_FullReport_FINAL.pdf

[CR46] Kamya PZ, Byrne M, Graba-Landry A, Dworjanyn SA (2016) Near-future ocean acidification enhances the feeding rate and development of the herbivorous juveniles of the crown-of-thorns starfish, *Acanthaster planci*. Coral Reefs 35:1241–1251. 10.1007/s00338-016-1480-6 10.1007/s00338-016-1480-6

[CR47] Karelitz S, Lamare MD, Mos B, De Bari H, Dworjanyn SA, Byrne M (2019) Impact of growing up in a warmer, lower pH future on offspring performance: transgenerational plasticity in a pantropical sea urchin. Coral Reefs 38:1085–1095. 10.1007/s00338-019-01855-z 10.1007/s00338-019-01855-z

[CR48] Kim JH, Lee HM, Cho YG, Shin JS, Yoo JW, Hong HK, Choi KS (2022) Effects of spawning stress on the immune capacity of blood cockle *Tegillarca granosa* occurring on the south coast of Korea. Fish Shellfish Immunol 120:15–22. 10.1016/j.fsi.2021.11.013 10.1016/j.fsi.2021.11.013 34774731

[CR49] Lesser MP (2006) Oxidative stress in marine environments: biochemistry and physiological ecology. Annu Rev Physiol 68:253–278. 10.1146/annurev.physiol.68.040104.110001 16460273 10.1146/annurev.physiol.68.040104.110001

[CR50] Lewis C, Ellis RP, Vernon E, Elliot K, Newbatt S, Wilson RW (2016) Ocean acidification increases copper toxicity differentially in two key marine invertebrates with distinct acid-base responses. Sci Rep 6:1–10. 10.1038/srep21554 26899803 10.1038/srep21554 PMC4761931

[CR51] Lucey NM, Lombardi C, DeMarchi L, Schulze A, Gambi MC, Calosi P (2015) To brood or not to brood: Are marine invertebrates that protect their offspring more resilient to ocean acidification? Sci Rep 5:12009. 10.1038/srep12009 26156262 10.1038/srep12009 PMC4648422

[CR52] Machado AM, Fernández-Boo S, Nande M, Pinto R, Costas B, Castro LFC (2022) The male and female gonad transcriptome of the edible sea urchin, *Paracentrotus lividus*: identification of sex-related and lipid biosynthesis genes. Aquaculture Reports 22:100936. 10.1016/j.aqrep.2021.100936 10.1016/j.aqrep.2021.100936

[CR53] Marčeta T, Matozzo V, Alban S, Badocco D, Pastore P, Marin MG (2020) Do males and females respond differently to ocean acidification? An experimental study with the sea urchin *Paracentrotus lividus*. Environ Sci Pollut Res 27:39516–39530. 10.1007/s11356-020-10040-7 10.1007/s11356-020-10040-7 PMC7524842 32651777

[CR54] Marčeta T, Locatello L, Alban S, Hassan MSA, Azmi N-NNM, Finos L, Badocco D, Marin MG (2022) Transgenerational effects and phenotypic plasticity in sperm and larvae of the sea urchin *Paracentrotus lividus* under ocean acidification. Aquat Toxicol 248:106208. 10.1016/j.aquatox.2022.106208 35635983 10.1016/j.aquatox.2022.106208

[CR55] Marisa I, Matozzo V, Munari M, Binelli A, Parolini M, Martucci A, Franceschinis E, Brianese N, Marin MG (2016) In vivo exposure of the marine clam *Ruditapes philippinarum* to zinc oxide nanoparticles: responses in gills, digestive gland and haemolymph. Environ Sci Pollut Res 23:15275–15293. 10.1007/s11356-016-6690-5 10.1007/s11356-016-6690-5 27102620

[CR56] Matozzo V, Costa Devoti A, Marin MG (2012a) Immunotoxic effects of triclosan in the clam *Ruditapes philippinarum*. Ecotoxicology 21:66–74. 10.1007/s10646-011-0766-2 21847659 10.1007/s10646-011-0766-2

[CR57] Matozzo V, Formenti A, Donadello G, Marin MG (2012b) A multi-biomarker approach to assess effects of Triclosan in the clam *Ruditapes philippinarum*. Mar Environ Res 74:40–46. 10.1016/j.marenvres.2011.12.002 22212174 10.1016/j.marenvres.2011.12.002

[CR58] Mecocci P, Fanó G, Fulle S, MacGarvey U, Shinobu L, Polidori MC, Cherubini A, Vecchiet J, Senin U, Beal MF (1999) Age-dependent increases in oxidative damage to DNA, lipids, and proteins in human skeletal muscle. Free Rad Biol Med 26:303–308. 10.1016/S0891-5849(98)00208-1 9895220 10.1016/S0891-5849(98)00208-1

[CR59] Migliaccio O, Pinsino A, Maffioli E, Smith AM, Agnisola C, Matranga V, Nonnis S, Tedeschi G, Byrne M, Gambi MC, Palumbo A (2019) Living in future ocean acidification, physiological adaptive responses of the immune system of sea urchins resident at a CO_2_ vent system. Sci Total Environ 672:938–950. 10.1016/j.scitotenv.2019.04.005 30981169 10.1016/j.scitotenv.2019.04.005

[CR60] Millero FJ (1995) Thermodynamics of the carbon dioxide system in the oceans. Geochim Cosmochim Acta 59(4):661–677. 10.1016/0016-7037(94)00354-O 10.1016/0016-7037(94)00354-O

[CR61] Millero FJ, Graham TB, Huang F, Bustos-Serrano H, Pierrot D (2006) Dissociation constants of carbonic acid in seawater as a function of salinity and temperature. Mar Chem 100:80–94. 10.1016/j.marchem.2005.12.001 10.1016/j.marchem.2005.12.001

[CR62] Moreira A, Figueira E, Soares AMVM, Freitas R (2016) The effects of arsenic and seawater acidification on antioxidant and biomineralization responses in two closely related Crassostrea species. Sci Total Environ 545–546:569–581. 10.1016/j.scitotenv.2015.12.029 10.1016/j.scitotenv.2015.12.029 26760276

[CR63] Morley SA, Suckling CC, Clark MS, Cross EL, Peck LS (2016) Long-term effects of altered pH and temperature on the feeding energetics of the Antarctic sea urchin, *Sterechinus neumayeri*. Biodiversity 17:34–45. 10.1080/14888386.2016.1174956 10.1080/14888386.2016.1174956

[CR64] Munari M, Chemello G, Finos L, Ingrosso G, Giani M, Marin MG (2016) Coping with seawater acidification and the emerging contaminant diclofenac at the larval stage: a tale from the clam *Ruditapes philippinarum*. Chemosphere 160:293–302. 10.1016/j.chemosphere.2016.06.095 27391052 10.1016/j.chemosphere.2016.06.095

[CR65] Munari M, Matozzo V, Gagné F, Chemello G, Riedl V, Finos L, Pastore P, Badocco D, Marin MG (2018) Does exposure to reduced pH and diclofenac induce oxidative stress in marine bivalves? A comparative study with the mussel *Mytilus galloprovincialis* and the clam *Ruditapes philippinarum*. Environ Pollut 240:925–937. 10.1016/j.envpol.2018.05.005 29949844 10.1016/j.envpol.2018.05.005

[CR66] Munari M, Matozzo V, Chemello G, Riedl V, Pastore P, Badocco D, Marin MG (2019) Seawater acidification and emerging contaminants: a dangerous marriage for haemocytes of marine bivalves. Environ Res 175:11–21. 10.1016/j.envres.2019.04.032 31100511 10.1016/j.envres.2019.04.032

[CR67] Munari M, Matozzo V, Benetello G, Riedl V, Pastore P, Badocco D, Marin MG (2020a) Exposure to decreased pH and caffeine affects hemocyte parameters in the mussel *Mytilus galloprovincialis*. J Mar Sci Eng 8:238. 10.3390/jmse8040238 10.3390/jmse8040238

[CR68] Munari M, Matozzo V, Riedl V, Pastore P, Badocco D, Marin MG (2020b) EAT BREATHE EXCRETE REPEAT: physiological responses of the mussel *Mytilus galloprovincialis* to diclofenac and ocean acidification. J Mar Sci Eng 8:907. 10.3390/jmse8110907 10.3390/jmse8110907

[CR69] Munari M, Devigili A, dalle Palle G, Asnicar D, Pastore P, Badocco D, Marin MG (2022) Ocean acidification, but not environmental contaminants, affects fertilization success and sperm motility in the sea urchin *Paracentrotus lividus*. J Mar Sci Eng 10:247. 10.3390/jmse10020247 10.3390/jmse10020247

[CR70] Oksanen J, Blanchet FG, Friendly M, Kindt R, Legendre P, McGlinn D, Minchin PR, O’Hara RB, Simpson GL, Solymos P, Stevens MHH, Szoecs E, Wagner H (2019) Vegan: community ecology package. R package version 2.5-2. https://cran.r-project.org/web/packages/vegan/vegan.pdf

[CR71] Orr JC, Fabry VJ, Aumont O, Bopp L, Doney SC, Feely RA, Gnanadesikan A, Gruber N, Ishida A, Joos F, Key RM, Lindsay K, Maier-Reimer E, Matear R, Monfray P, Mouchet A, Najjar RG, Plattner GK, Rodgers KB, Sabine CL, Sarmiento JL, Schlitzer R, Slater RD, Totterdell IJ, Weirig MF, Yamanaka Y, Yool A (2005) Anthropogenic ocean acidification over the twenty-first century and its impact on calcifying organisms. Nature 437:681–686. 10.1038/nature04095 16193043 10.1038/nature04095

[CR72] Palombo C, Chiarore A, Ciscato M, Asnicar D, Mirasole A, Fabbrizzi E, Teixido N, Munari M (2023) Thanks mum. Maternal effects in response to ocean acidification of sea urchin larvae at different ecologically relevant temperatures. Mar Pollut Bull 188:114700. 10.1016/j.marpolbul.2023.114700 36773584 10.1016/j.marpolbul.2023.114700

[CR73] Pastore A, Badocco D, Cappellin L, Tubiana M, Zanut A, Bogialli S, Roverso M, Pastore P (2024) Accurate pH monitoring of highly concentrated saline aqueous solutions (seawater-like) with a pH colorimetric sensor array. ACS Sensors 9(3):1482–1488. 10.1021/acssensors.3c02585 38416572 10.1021/acssensors.3c02585

[CR74] Paterno M, Schiavina M, Aglieri G, Souissi JB, Boscari E, Casagrandi R, Chassanite A, Chiantore M, Congiu L, Guarnieri G, Kruschel C, Macic V, Marino IAM, Papetti C, Patarnello T, Zane L, Melià P (2017) Population genomics meet Lagrangian simulations: oceanographic patterns and long larval duration ensure connectivity among *Paracentrotus lividus* populations in the Adriatic and Ionian seas. Ecol Evol 7:2463–2479. 10.1002/ece3.2844 28428839 10.1002/ece3.2844 PMC5395429

[CR75] Pörtner HO, Langenbuch M, Michaelidis B (2005) Synergistic effects of increased CO_2_, temperature and hypoxia on marine animals. J Geophys Res-Oceans 110:1–15. 10.1029/2004JC002561 10.1029/2004JC002561

[CR76] Pörtner HO, Langenbuch M, Reipschläger A (2004) Biological impact of elevated ocean CO_2_ concentrations: lessons from animal physiology and earth history. J Oceanogr 60:705–718

[CR77] R Core Team (2021) R: A language and environment for statistical computing. R Foundation for Statistical Computing, Vienna

[CR78] Raven J, Caldeira K, Elderfield H, Hoegh-Guldberg O, Liss P, Riebesell U, Shepherd J, Turley C, Watson A (2005) Ocean acidification due to increasing atmospheric carbon dioxide. The Royal Society, London

[CR79] Régis MB (1979) Analyse des fluctuations des indices physiologiques chez deux echinoids (*Paracentrotus lividus* (Lmk) et *Arbacia lixula* (L.) du Golfe de Marseille. Tethys 9:167–181

[CR80] Sabine CL, Feely RA, Gruber N, Key RM, Lee K, Bullister JL, Wanninkhof R, Wong CS, Wallace DWR, Tilbrook B, Millero FJ, Peng TH, Kozyr A, Ono T, Rios AF (2004) The oceanic sink for anthropogenic CO_2_. Science 305:367–371. 10.1126/science.1097403 15256665 10.1126/science.1097403

[CR81] Sartori D, Pellegrin D, Macchia S, Gaion A (2016) Can echinoculture be a feasible and effective activity? Analysis of fast reliable breeding conditions to promote gonadal growth and sexual maturation in *Paracentrotus lividus*. Aquaculture 451:39–46. 10.1016/j.aquaculture.2015.08.037 10.1016/j.aquaculture.2015.08.037

[CR82] Schäfer S, Abele D, Weihe E, Köhler A (2011) Sex-specific biochemical and histological differences in gonads of sea urchins (*Psammechinus miliaris*) and their response to phenanthrene exposure. Mar Environ Res 71:70–78. 10.1016/j.marenvres.2010.10.004 21094999 10.1016/j.marenvres.2010.10.004

[CR83] Shetye SS, Naik H, Kurian S, Shenoy D, Kuniyil N, Fernandes M, Hussain A (2020) pH variability off Goa (eastern Arabian Sea) and the response of sea urchin to ocean acidification scenarios. Mar Ecol 41:1–11. 10.1111/maec.12614 10.1111/maec.12614

[CR84] Shpigel M, McBride SC, Marciano S, Lupatsch I (2004) The effect of photoperiod and temperature on the reproduction of European sea urchin *Paracentrotus lividus*. Aquaculture 232:343–355. 10.1016/S0044-8486(03)00539-8 10.1016/S0044-8486(03)00539-8

[CR85] Smith IK, Vierheller TL, Thorne CA (1988) Assay of glutathione reductase in crude tissue homogenates using 5,5′-dithiobis(2-nitrobenzoic acid). Anal Biochem 175:408–413. 10.1016/0003-2697(88)90564-7 3239770 10.1016/0003-2697(88)90564-7

[CR86] Sui Y, Hu M, Shang Y, Wu F, Huang X, Dupont S, Storch D, Pörtner H-O, Li J, Lu W, Wang Y (2017) Antioxidant response of the hard shelled mussel *Mytilus coruscus* exposed to reduced pH and oxygen concentration. Ecotoxicol Environ Saf 137:94–102. 10.1016/j.ecoenv.2016.11.023 27915148 10.1016/j.ecoenv.2016.11.023

[CR87] Sun T, Tang X, Jiang Y, Wang Y (2017) Seawater acidification induced immune function changes of haemocytes in *Mytilus edulis*: a comparative study of CO_2_ and HCl enrichment. Sci Rep 7:1–10. 10.1038/srep41488 28165002 10.1038/srep41488 PMC5292689

[CR88] Taylor JR, Lovera C, Whaling PJ, Buck KR, Pane EF, Barry JP (2014) Physiological effects of environmental acidification in the deep-sea urchin *Strongylocentrotus fragilis*. Biogeosciences 11:1413–1423. 10.5194/bg-11-1413-2014 10.5194/bg-11-1413-2014

[CR89] Thor P, Oliva EO (2015) Ocean acidification elicits different energetic responses in an Arctic and a boreal population of the copepod *Pseudocalanus acuspes*. Mar Biol 162:799–807. 10.1007/s00227-015-2625-9 10.1007/s00227-015-2625-9

[CR90] Thor P, Bailey A, Dupont S, Calosi P, Søreide JE, De Wit P, Guscelli E, Loubet-Sartrou L, Deichmann IM, Candee MM, Svensen C, King AL, Bellerby RGJ (2018) Contrasting physiological responses to future ocean acidification among Arctic copepod populations. Glob Change Biol 24:e365–e377. 10.1111/gcb.13870 10.1111/gcb.13870 28816385

[CR91] Uthicke S, Patel F, Karelitz S, Luter H, Webster N, Lamare M (2020) Key biological responses over two generations of the sea urchin *Echinometra* sp. A under future ocean conditions. Mar Ecol Prog Ser 637:87–101. 10.3354/meps13236 10.3354/meps13236

[CR92] Vargas CA, Lagos NA, Lardies MA, Duarte C, Manríquez PH, Aguilera VM, Broitman B, Widdicombe S, Dupont S (2017) Species-specific responses to ocean acidification should account for local adaptation and adaptive plasticity. Nat Ecol Evol. 10.1038/s41559-017-0084 10.1038/s41559-017-0084 28812677

[CR93] Vargas CA, Cuevas LA, Broitman BR, San Martin VA, Lagos NA, Gaitán-Espitia JD, Dupont S (2022) Upper environmental *p*CO_2_ drives sensitivity to ocean acidification in marine invertebrates. Nat Clim Chang. 10.1038/s41558-021-01269-2 10.1038/s41558-021-01269-2

[CR94] Waldbusser GG, Hales B, Langdon CJ, Haley BA, Schrader P, Brunner EL, Gray MW, Miller CA, Gimenez I, Hutchinson G (2015) Ocean acidification has multiple modes of action on bivalve larvae. PLoS ONE 10(6): e0128376. 10.1371/journal.pone.0128376 10.1371/journal.pone.0128376 PMC4465621 26061095

[CR95] Wang Q, Cao R, Ning X, You L, Mu C, Wang C, Wei L, Cong M, Wu H, Zhao J (2016) Effects of ocean acidification on immune responses of the Pacific oyster *Crassostrea gigas*. Fish Shellfish Immunol 49:24–33. 10.1016/j.fsi.2015.12.025 26706224 10.1016/j.fsi.2015.12.025

[CR96] Wu LT, Chu KH (2010) Characterization of an ovary-specific glutathione peroxidase from the shrimp *Metapenaeus ensis* and its role in crustacean reproduction. Comp Biochem Physiol B: Biochem Mol Biol 155(1):26–33. 10.1016/j.cbpb.2009.09.005 19788927 10.1016/j.cbpb.2009.09.005

