# Supplementary material for: Investigating intraspecific variability in the biological responses of sea urchins (Paracentrotus lividus) to seawater acidification
Source: Environ Sci Pollut Res Int. 2024 Aug 9;31(39):51687–701. doi: 10.1007/s11356-024-34618-7 (PMC11374922; doi:10.1007/s11356-024-34618-7)
Supplement: Supplementary file 1 — Supplementary file1 (DOCX 751 KB) [file 11356_2024_34618_MOESM1_ESM.docx]

**Figure S1.** Location of sampling site LS in the Lagoon of Venice and sampling site MS in the Gulf of Trieste. Modified from Asnicar et al., 2021 – MERE, 169 - <https://doi.org/10.1016/j.marenvres.2021.105372>

**
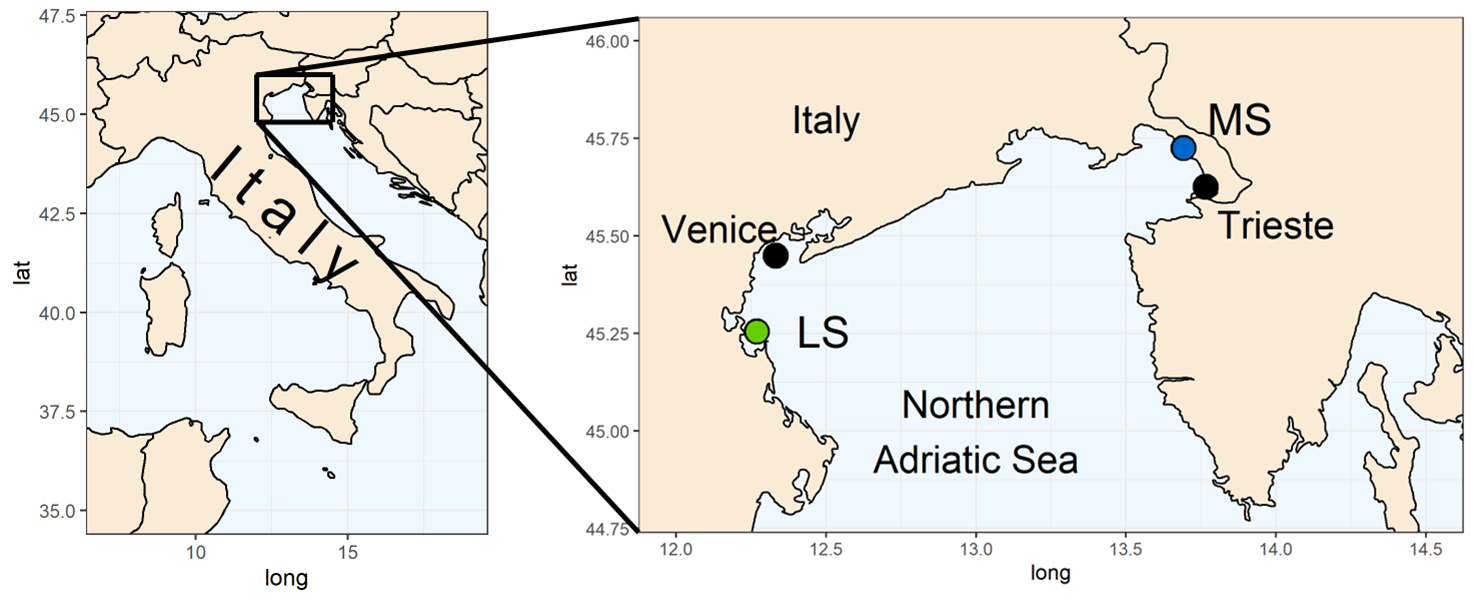
**

**Table S1.** Average monthly values ± standard deviation of the physico-chemical parameters measured at Site 1 and Site 2 from 2009 to 2020.

Temperature, salinity, pH and dissolved oxygen (DO) data of LS were obtained from Hydrobiological Station of the Department of Biology (University of Padova) <https://chioggia.biologia.unipd.it/en/the-database/parameters-of-lagoon/>

Chlorophyll a (Chl a) data of LS (Lagoon Site) and all the data of MS (Marine Site) were obtained from E.U. Copernicus Marine Service Information (CMEMS) <https://marine.copernicus.eu/>

For each parameter the coefficient of variation (CV) is reported.

| Site | Month | Temperature (°C) | CV  T °C | Salinity | CV  salinity | pH_T_ | CV  pH | Dissolved oxygen (µM) | CV  DO | Chlorophyll a  (mg m^-3^) | CV  Chl a |
| --- | --- | --- | --- | --- | --- | --- | --- | --- | --- | --- | --- |
| LS | 01 | 8.6 ± 1.4 | 16.0 | 34 ± 1.4 | 4.1 | 8.2 ± 0.1 | 1.2 | 247.3 ± 39.9 | 16.1 | 3.4 ± 1.2 | 34.4 |
| LS | 02 | 8.5 ± 2.1 | 25.0 | 33.3 ± 2.6 | 7.8 | 8.2 ± 0.1 | 1.0 | 253 ± 35.6 | 14.1 | 3.6 ± 0.8 | 23.6 |
| LS | 03 | 10.9 ± 1.6 | 14.3 | 33.3 ± 2.1 | 6.2 | 8.2 ± 0.1 | 1.2 | 242.6 ± 32.8 | 13.5 | 4.2 ± 0.4 | 10.7 |
| LS | 04 | 15.3 ± 0.7 | 4.5 | 33.1 ± 2.2 | 6.7 | 8.3 ± 0.1 | 0.9 | 216.1 ± 23.5 | 10.9 | 5 ± 2.9 | 59.2 |
| LS | 05 | 19.2 ± 1.3 | 6.9 | 32.6 ± 2 | 6.2 | 8.2 ± 0.1 | 1.6 | 198.2 ± 23.7 | 12.0 | 8.2 ± NA | NA |
| LS | 06 | 23.8 ± 1 | 4.1 | 32.8 ± 1.4 | 4.2 | 8.3 ± 0.1 | 1.3 | 171.8 ± 15.5 | 9.0 | 5.5 ± 0.9 | 17.0 |
| LS | 07 | 26.3 ± 0.7 | 2.6 | 33.9 ± 1.7 | 5.0 | 8.2 ± 0.1 | 1.5 | 146.5 ± 19 | 12.9 | 6.7 ± 3.4 | 50.1 |
| LS | 08 | 26.4 ± 0.8 | 3.1 | 34.1 ± 1.6 | 4.6 | 8.2 ± 0.1 | 1.1 | 152.1 ± 15.4 | 10.1 | 4.7 ± 1.4 | 30.5 |
| LS | 09 | 23 ± 1.1 | 4.7 | 34.2 ± 1.4 | 4.1 | 8.2 ± 0.1 | 1.2 | 168.6 ± 13.4 | 8.0 | 4.5 ± 1.6 | 36.3 |
| LS | 10 | 18.4 ± 0.8 | 4.6 | 34.5 ± 1.2 | 3.4 | 8.2 ± 0.1 | 1.0 | 185 ± 16.5 | 8.9 | 3.2 ± 1.9 | 57.6 |
| LS | 11 | 14.4 ± 0.8 | 5.6 | 33.4 ± 1.7 | 5.0 | 8.2 ± 0.1 | 1.2 | 210 ± 24 | 11.4 | 3.5 ± 2.5 | 72.0 |
| LS | 12 | 10.7 ± 1.2 | 11.2 | 33.9 ± 1.9 | 5.6 | 8.2 ± 0.1 | 1.2 | 235.7 ± 27.9 | 11.8 | 3.4 ± 1.2 | 34.3 |
|  |  |  |  |  |  |  |  |  |  |  |  |
| MS | 01 | 9.3 ± 0.8 | 9.1 | 37.2 ± 0.5 | 1.3 | 8.2 ± 0 | 0.5 | 254 ± 7.8 | 3.1 | 1 ± 0.3 | 31.0 |
| MS | 02 | 8.3 ± 1.2 | 14.6 | 37.3 ± 0.4 | 1.1 | 8.2 ± 0 | 0.5 | 265.9 ± 8 | 3.0 | 0.7 ± 0.3 | 39.1 |
| MS | 03 | 9.6 ± 1.1 | 11.2 | 37.3 ± 0.3 | 0.8 | 8.2 ± 0 | 0.4 | 269 ± 9.1 | 3.4 | 0.8 ± 0.2 | 29.9 |
| MS | 04 | 13.1 ± 0.7 | 5.5 | 37 ± 0.5 | 1.2 | 8.2 ± 0 | 0.4 | 262 ± 7.6 | 2.9 | 1.2 ± 0.3 | 23.2 |
| MS | 05 | 17.8 ± 0.8 | 4.4 | 36.5 ± 0.9 | 2.4 | 8.1 ± 0 | 0.5 | 242.9 ± 11 | 4.5 | 2 ± 0.8 | 40.4 |
| MS | 06 | 22.6 ± 0.6 | 2.6 | 36.4 ± 0.5 | 1.4 | 8 ± 0.1 | 0.7 | 229.7 ± 17.6 | 7.6 | 1.7 ± 1.7 | 98.5 |
| MS | 07 | 25.3 ± 0.5 | 2.0 | 37 ± 0.8 | 2.1 | 8 ± 0 | 0.4 | 217.2 ± 17.6 | 8.1 | 1.2 ± 0.9 | 75.1 |
| MS | 08 | 25.9 ± 0.6 | 2.2 | 37.5 ± 0.6 | 1.7 | 8 ± 0 | 0.2 | 203.4 ± 17.9 | 8.8 | 1 ± 0.7 | 66.1 |
| MS | 09 | 23.5 ± 0.6 | 2.4 | 37.3 ± 0.5 | 1.4 | 8 ± 0 | 0.3 | 196.5 ± 15.2 | 7.8 | 1.4 ± 0.7 | 49.5 |
| MS | 10 | 19.5 ± 0.7 | 3.8 | 37 ± 0.6 | 1.7 | 8.1 ± 0 | 0.5 | 206.5 ± 12.8 | 6.2 | 1.5 ± 0.5 | 34.0 |
| MS | 11 | 15.4 ± 1 | 6.2 | 36.7 ± 0.5 | 1.4 | 8.1 ± 0.1 | 0.6 | 222.2 ± 4.8 | 2.2 | 1.5 ± 0.4 | 24.1 |
| MS | 12 | 11.8 ± 0.8 | 7.0 | 37 ± 0.4 | 1.0 | 8.2 ± 0 | 0.6 | 239.9 ± 7.3 | 3.0 | 1.1 ± 0.2 | 15.8 |


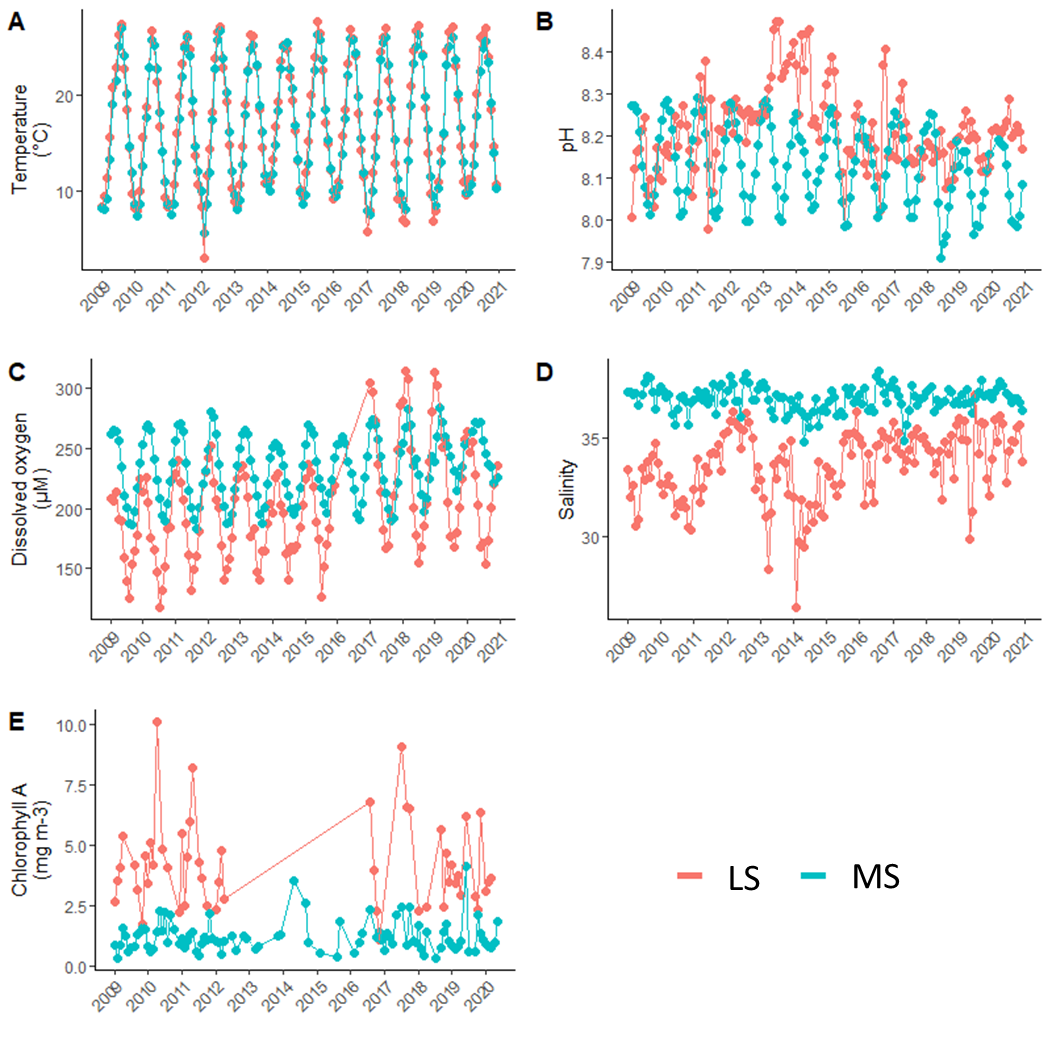


**Figure S2.** Time series graphs of the physico-chemical parameters measured at LS (Lagoon Site) and MS (Marine Site) from 2009 to 2020.

Temperature, salinity, pH and dissolved oxygen data of LS were obtained from Hydrobiological Station of the Department of Biology (University of Padova) <https://chioggia.biologia.unipd.it/en/the-database/parameters-of-lagoon/>

Chlorophyll-A data of LS and all the data of MS were obtained from E.U. Copernicus Marine Service Information (CMEMS) <https://marine.copernicus.eu/>

**Table S2.** Results of Levene test for homogeneity of variance to compare the variability of five parameter selected between LS (Lagoon Site) and MS (Marine Site).

The variance is significantly different between sites for salinity, dissolved oxygen and chlorophyll a.

| **Parameter** | **F value** | ***p* value** |
| --- | --- | --- |
| **Temperature (°C)** | 0.133 | 0.716 |
| **Salinity** | 93.548 | < 0.001 |
| **pH** | 1.032 | 0.311 |
| **Dissolved oxygen (µM)** | 22.220 | < 0.001 |
| **Chlorophyll A (mg/m^3^)** | 37.847 | < 0.001 |

**Table S3.** Average values ± SD of the seawater parameters recorded during the experiment. S = Salinity, TA = total alkalinity; DIC = total dissolved inorganic carbon; *p*CO_2_ = CO_2_ partial pressure; Ωca = calcite saturation state; Ωar = aragonite saturation state. Modified from Asnicar et al., 2021 – MERE, 169 - <https://doi.org/10.1016/j.marenvres.2021.105372>

| **Origin site** | **Nominal pH** | **pH_T_** | **T °C** | **S** | **[O_2_] ppm** | **TA** | **DIC** | ***p*CO_2_** | **Ωca** | **Ωar** |
| --- | --- | --- | --- | --- | --- | --- | --- | --- | --- | --- |
| ***LS*** | *pH Amb* | 8.03 ± 0.07 | 18.24 ± 1.25 | 34.86 ± 1.34 | 6.92 ± 0.83 | 2868.51 ± 57.02 | 2674.70 ± 70.78 | 713.16 ± 170.90 | 5.09 ± 0.82 | 3.29 ± 0.54 |
|  | *pH -0.4* | 7.61 ± 0.12 | 18.31 ± 1.29 | 34.84 ± 1.34 | 6.76 ± 0.81 | 2879.95 ± 62.61 | 2851.54 ± 62.60 | 2129.69 ± 385.54 | 2.08 ± 0.37 | 1.35 ± 0.24 |
| ***MS*** | *pH Amb* | 8.04 ± 0.05 | 18.72 ± 0.71 | 34.53 ± 2.04 | 6.81 ± 0.64 | 2910.50 ± 66.92 | 2707.26 ± 72.72 | 674.21 ± 95.69 | 5.32 ± 0.56 | 3.44 ± 0.37 |
|  | *pH -0.4* | 7.64 ± 0.07 | 18.70 ± 0.73 | 34.54 ± 2.00 | 6.74 ± 0.63 | 2926.57 ± 74.09 | 2881.08 ± 78.75 | 1885.10 ± 308.55 | 2.35 ± 0.36 | 1.52 ± 0.23 |

Alkalinity and related seawater carbonate system parameters of Ambient pH condition (pH Amb in Table S3) are within the natural range of variability for the Lagoon of Venice.

In the work of Faganeli et al. (2021) alkalinity ranges between 2600 to 3100 were found on samples from the Venetian lagoon. Also in the work of Ingrosso et al. (2016) the alkalinity values showed seasonal excursions with peaks up to 2933 μmol kg−1 due (according to the authors) to the proximity to the mouth of the Isonzo (4800 μmol kg−1).

However, it is possible that the high alkalinity values are due to the proximity of the sampling site to the mouth of the Brenta (4500 μmol kg−1) (Giani et al., 2023).

Faganeli J., Ogrinc N., Tamše S., Krajnc B, Turk V., Malej A., Kovač N., 2021. »Kisanje« severnega Jadrana. Acta Chim. Slov. 68, S87–S93. DOI: 10.17344/acsi.2021.7002

Ingrosso G., Giani M., Cibic T., Karuza A., Kralj M., Del Negro P., 2016. Carbonate chemistry dynamics and biological processes along a river–sea gradient (Gulf of Trieste, northern Adriatic Sea). Journal of Marine Systems 155 35–49.

Giani M., Ogrinc N., Tamše S., Cozzi S., 2023. Elevated River Inputs of the Total Alkalinity and Dissolved Inorganic Carbon in the Northern Adriatic Sea. Water, 15, 894 https://doi.org/10.3390/w15050894
